# Complete loss of *TP53* and *RB1* is associated with complex genome and low immune infiltrate in pleomorphic rhabdomyosarcoma

**DOI:** 10.1016/j.xhgg.2023.100224

**Published:** 2023-07-19

**Authors:** Hannah C. Beird, Chia-Chin Wu, Michael Nakazawa, Davis Ingram, Joseph R. Daniele, Rossana Lazcano, Latasha Little, Christopher Davies, Najat C. Daw, Khalida Wani, Wei-Lien Wang, Xingzhi Song, Curtis Gumbs, Jianhua Zhang, Brian Rubin, Anthony Conley, Adrienne M. Flanagan, Alexander J. Lazar, P. Andrew Futreal

**Affiliations:** 1 Department of Genomic Medicine, The University of Texas MD Anderson Cancer Center, Houston, TX 77030, USA; 2 Department of Cancer Medicine, The University of Texas MD Anderson Cancer Center, Houston, TX 77030, USA; 3 Department of Translational and Molecular Pathology, The University of Texas MD Anderson Cancer Center, Houston, TX 77030, USA; 4 TRACTION Platform, Division of Therapeutics Discovery, The University of Texas MD Anderson Cancer Center, Houston, TX 77030, USA; 5 Research Department of Pathology, UCL Cancer Institute, London WC1E 6DD, UK; 6 Department of Pediatrics, The University of Texas MD Anderson Cancer Center, Houston, TX 77030, USA; 7 Department of Pathology, The University of Texas MD Anderson Cancer Center, Houston, TX 77030, USA; 8 Institute Chair, Cleveland Clinic, Cleveland, OH 44195, USA; 9 Department of Sarcoma Medical Oncology, The University of Texas MD Anderson Cancer Center, Houston, TX 77030, USA; 10 Royal National Orthopaedic Hospital NHS Trust, Stanmore, Middlesex HA7 4LP, UK

**Keywords:** Rhabdomyosarcoma, Sarcoma, TP53, Complex karyotype, Pleomorphic rhabdomyosarcoma

## Abstract

Rhabdomyosarcoma accounts for roughly 1% of adult sarcomas, with pleomorphic rhabdomyosarcoma (PRMS) as the most common subtype. Survival outcomes remain poor for patients with PRMS, and little is known about the molecular drivers of this disease. To better characterize PRMS, we performed a broad array of genomic and immunostaining analyses on 25 patient samples. In terms of gene expression and methylation, PRMS clustered more closely with other complex karyotype sarcomas than with pediatric alveolar and embryonal rhabdomyosarcoma. Immune infiltrate levels in PRMS were among the highest observed in multiple sarcoma types and contrasted with low levels in other rhabdomyosarcoma subtypes. Lower immune infiltrate was associated with complete loss of both *TP53* and *RB1*. This comprehensive characterization of the genetic, epigenetic, and immune landscape of PRMS provides a roadmap for improved prognostications and therapeutic exploration.

## Teaser

Pleomorphic rhabdomyosarcoma (PRMS) is a soft-tissue tumor of the skeletal muscle. The genomic and transcriptomic profiles of our PRMS cohort were more similar to other complex karyotype sarcomas than to pediatric alveolar and embryonal rhabdomyosarcoma. Those with lower immune infiltrate levels were associated with complete loss of *TP53* and *RB1*.

## Introduction

Rhabdomyosarcomas are soft-tissue tumors that exhibit skeletal muscle-type differentiation. While alveolar rhabdomyosarcoma (ARMS) (MIM: 268220) and embryonal rhabdomyosarcoma (ERMS) occur most frequently in children, pleomorphic rhabdomyosarcoma (PRMS) is the most common subtype in adults.^1^ Between 1973 and 2014, PRMS accounted for only 462 of 4,787 (9.7%) rhabdomyosarcomas documented in the Surveillance, Epidemiology, and End Results (SEER) database.^2^ PRMS has a slight male predominance (1.8:1) and an average age of diagnosis of 40–50 years and arises most commonly in the extremities.^1^^,^^3^ The overall survival (OS) for PRMS is worse than for ARMS and ERMS,^4^^,^^5^ and the median OS is worse for PRMS than for many other high-grade adult soft-tissue sarcomas,^3^^,^^6^ with a 5-year OS rate of only 26%.^2^ For localized PRMS, wide surgical resection remains the primary treatment, as these tumors have poor response rates to common rhabdomyosarcoma-specific chemotherapies.^3^ However, even with surgery, disease progression is common, and there is a high propensity for metastasis, especially to the lungs.

Diagnosis of PRMS can be challenging because of varied histologic patterns of PRMS and similarity in immunohistochemical staining between PRMS and other rhabdomyosarcoma subtypes.^7^ In ARMS, pathognomonic PAX3-FOXO1/PAX7-FOXO1 fusions are found in a majority of cases with few other somatic mutations, and these fusions are absent in PRMS. ERMS has alterations in *KRAS*, *NRAS*, and *NF1*, which are infrequent in PRMS.^8^ Both ARMS and ERMS have simple karyotypes, whereas those of PRMS are complex, often harboring deleterious alterations in *TP53*, *RB1*, and *NF1*.^9^^,^^10^^,^^11^ Copy-number profiles of PRMS show levels of genomic instability reminiscent of those in adult/non-translocation-driven sarcomas such as osteosarcoma.^12^ Gains in 1p, 18q, and 20p and losses in 3p, 5q, 10q, 13, and 15q have been seen.^12^ One case report showed hypertriploidy by spectral karyotyping^10^ and another patient-derived cell line with a complex pseudotetraploid karyotype.^13^ Although the processes driving PRMS are unclear, case reports suggest that germline mutations in *TP53* and mismatch repair genes *PMS2*, *MSH2*, and *MLH1* can be predisposing factors.^14^^,^^15^^,^^16^^,^^17^

Given the relatively poor outcomes for patients with PRMS and its chemotherapy-resistant nature, further characterization of the genomic, epigenetic, and immune landscape is necessary to provide the basis for alternative treatment strategies. In this study, we conducted high-depth whole-genome, whole-exome, and bulk transcriptome sequencing, methylation array analysis, T cell receptor beta sequencing, multiplex immunofluorescence staining for immune infiltrates, and tertiary lymphoid structure phenotyping to delineate the molecular architecture of PRMS.

## Materials and methods

### Patient cohort

Patients were collected at three institutions. Patients at the Royal National Orthopedic Hospital (RNOH) consented to sample collection as described.^18^ For the other institutions, samples were obtained by informed consent and with approval by each institutional review board. Patients from the MD Anderson Cancer Center (MDACC) were diagnosed between 1999 and 2020. All samples were rereviewed by expert sarcoma pathologists to ascertain the diagnosis. Clinical information that was collected included age at diagnosis, primary anatomical site, largest dimension of tumor size at diagnosis, metastatic disease at presentation, date of surgery (if performed), date of death if applicable, and last known follow-up. First-line chemotherapy administered was recorded when available (in patients from the MDACC). Frozen specimens of tumor and matching histologially normal tissue (adjacent or peripheral blood mononuclear cells) were used for genome, exome, and bulk transcriptome sequencing and methylation array analysis. Formalin-fixed, paraffin-embedded (FFPE) specimens were used for staining.

### Whole-genome sequencing

For MD Anderson samples, genomic DNA was extracted using the frozen tissue protocol from the QIAamp DNA Mini kit as described previously.^19^ Whole-genome sequencing (WGS) was performed to an average sequencing depth of 69× for tumors and of 30× for germline samples for 14 patients (frozen samples). Alignment against the hg19 reference was done with using the Burrows-Wheeler Aligner Maximal Exact Match (BWA-MEM). Somatic point mutations (MuTect and Pindel), copy-number alterations (HMMcopy), kataegis, chromothripsis, structural rearrangements (BRASS), genome doubling, and subclonal architecture were analyzed as described.^20^ The Wilcox test was used to compare point mutation burdens.

### Exome sequencing

Exomes with matching germline samples were generated for seven patients (3 frozen and 4 FFPE samples). SureSelect Human All Exon V4 (Agilent) library preparation was done and sequenced to a target depth of 200× with matched histologically normal samples at a target depth of 100×. Somatic point mutations were called as described above. Copy-number alterations were called with exomecn, and structural rearrangements were called with Delly^21^ and Lumpy.^22^

### RNA sequencing and gene expression analysis

RNA was extracted by homogenization in 1 mL Trizol reagent, phase separation with chloroform, and recovery of total RNA using Directzol columns (Zymo Research, Irvine, CA, USA). Samples with RNA Integrity Number (RIN) ≥7 were processed. RNA amounts were normalized, and NEB library average insert size was 317 bp targeted for 200 million read depth on Illumina on NextSeq 100 bp paired-end reads.

RNA sequencing reads were mapped to the hg19 reference genome using STAR aligner.^23^ To calculate gene expression, raw count data of each gene were obtained with HTSeq^24^ and normalized by scaling the raw library size using calcNormFactors in the edgeR package in R. Then, Voom transformation was applied to normalized counts, and a linear model was fit to the data for differential expression analysis using the Limma package.^25^ Significantly deregulated genes between any two groups were selected (p ≤ 0.05 and fold change ≥ 1.5). Pathway analyses of differentially expressed genes was performed using gene set enrichment analysis (GSEA)^26^ and the web-accessible program DAVID.^27^^,^^28^ Fusions that were detected by at least two tools were selected as described.^20^ In addition, we found that our groups of sarcoma samples were confounded by batch-associated factors, which made it impossible to separate the impacts of batches of groups using the traditional batch-effect-removal methods.^29^ Therefore, we used a rank-based method, single-sample GSEA, to compare the transcriptomes of our PRMS samples and samples of multiple other sarcoma histological subtypes (more details in the supplemental methods). To identify expressed neoantigens, we first applied Mutect^30^ to call point mutations from the aligned RNA sequencing (RNA-seq) file tumor samples against the WGS bam files of their paired histologically normal samples. We then integrated the predicted neoantigens from WGS data with the called mutations from RNA-seq data. We also integrated in-frame rearrangements and fusion transcripts to identify expressed rearrangements in each sample, whose genomic breakpoints detected from WGS data and fusion transcript junction regions detected from RNA-seq are in the same genic regions.

### Immune infiltration analysis

Immune infiltration scores were calculated from the gene expression data using ESTIMATE.^31^ Comparisons of the ESTIMATE scores between tumor types were done using the Wilcox test. Immune cell profiles of samples were generated using single-sample GSEA (ssGSEA) enrichment scores of 29 immune gene signatures.^26^

### Methylation array and analysis

Genomic DNA was extracted from either fresh frozen or FFPE tumor samples. Using 500 ng DNA, bisulfite conversion was conducted using the Zymo EZ DNA Methylation-Gold kit (Zymo Research), and then the DNA was hybridized against the EPIC bead chip arrays (Illumina, San Diego, CA, USA) by UCL Genomics. All bisulfite-converted FFPE samples were restored with the Infinium FFPE DNA Restore kit (Illumina). The minfi R package^32^ was used for quality control and pre-processing of raw DNA methylation files. The methylation data of other rhabdomyosarcoma samples^5^ were downloaded from the Gene Expression Omnibus and were processed using the same analyses. No significant batch effect was seen in our PRMS samples and these rhabdomyosarcoma samples.

### T cell receptor sequencing

Two replicate samples for each of the 18 patients with PRMS were sent to Adaptive Technologies for survey resolution of T cell receptor (TCR) beta sequencing using their ImmunoSEQ Assay. The ImmunoSEQ analyzer was used to obtain values for rearrangements, clonality, and entropy (richness). The other TCR datasets used for comparison were primary melanoma (N = 199),^33^ primary non-small cell lung cancer (N = 225),^34^ and osteosarcoma (N = 41),^20^ all available through the immuneACCESS database.

### Multiplex immunofluorescence

Each 4-μm-thick full tumor section was stained for nine markers: CD45RO for all immune cells (pure); CD3 epsilon for T cells (D7A6E; 1:100); CD8 for cytotoxic T cells (1:25); FOXP3 for regulatory T cells (1:50); Ki67 for proliferation (1:100); PD1 (1:250) and PD-L1 (1:500) for immune checkpoint; CD68 for monocyte/macrophage population (1:50); and DAPI for nuclear assessment. Details on procedures and area selection were previously published.^35^ Following standard segmentation and phenotyping with inform (Akoya Biosciences), the individual sample files were merged and consolidated using the Phenoptr Reports script in R (Akoya Biosciences). During the analysis in Phenoptr Reports, the number of coincidences of different combinations of phenotypes within a 15 μm radius were calculated, and these counts were then plotted using GraphPad PRISM 9 software. p values <0.05 were deemed significant.

### Immunohistochemistry for tertiary lymphoid structures

Immunohistochemistry was performed using a Leica Bond RXm automated stainer, using the Bond Refine Detection kit (Leica). Samples underwent antigen retrieval in citrate buffer at 100°C for 20 min and were incubated for 15 min with monoclonal mouse anti-human CD20 antibody, clone L26 (Dako, M075501-2), diluted 1:1,400 in Bond Primary Antibody Diluent (Leica). We considered positive CD20 when a group of at least 50 cells (lymphoid aggregates) showed CD20 positivity^36^ and negative CD20 when we either did not see CD20-positive cells or when we saw CD20 lymphocytes in a diffuse pattern (without forming aggregates).

### Statistical methods

To characterize the association of gene expression with age at diagnosis (Figure 4), the age at diagnosis as a continuous variable was compared against the normalized gene counts for *PAX3* and *DMD*, respectively, using the linear regression (lm) function in R, with r^2^ >0.4 deemed significant. To compare the total number of TCR rearrangements between tumor types (Figures 5C and 5D), two-sided t tests were performed (Figure 5C). TCR productive clonality shows the proportion of TCRs that produce in-frame sequences without stop codons, allowing for amino acids that are fully functional to recognize antigen. Two-sided t tests were used to compare these proportions between tumor types (Figure 5D). To examine any relationships between the immune infiltrate and the genomic characteristics (Figures 6E–6G), Pearson correlation analyses were performed using the ESTIMATE immune scores for each sample against copy-number log2 ratio scores or against normalized RNA gene counts. Survival analysis and Kaplan-Meir curves were generated using GraphPad Prism 9 software, using the log-rank test to compare survival times. p values <0.05 were considered significant.

## Results

Similar to historical cases of PRMS,^37^ the median age of our cohort was 58 years (range: 13–92 years), with the majority of primary sites in the extremity (12/23, 52%) and the primary tumor sizes ranging from 3.5 to 30 cm (Table 1). The median OS was 2.8 years for all patients (Figure S1). The difference in survival, based on the primary tumor size cutoff of 5 cm that is recommended for rhabdomyosarcoma staging, was 6.9 years in tumors less than 5 cm vs. 1.67 years for tumors greater than 5 cm (p = 0.067, hazard ratio [HR] 0.32, 95% confidence interval [CI] 0.1–1.08) (Figure S1).^38^ Similarly, patients with primary tumors in the extremities had an OS compared with patients with primary tumors in non-extremity sites of 6.9 vs. 1.9 years (p = 0.13, HR 0.43, 95% CI 0.07–1.44).

**Table 1:** Clinicopathologic features for patients with PRMS from RNOH and MDACC

No. patients	
RNOH	13
MDACC	10
Age (range 12–92), years	
Mean	58
Median	60
Primary tumor site	
Trunk	9
Extremity	12
Head and neck	2
Size (range 3.5–30 cm, 1 unknown), cm	
Mean	8.5
Median	7.15
Chemotherapy prior to biopsy	
No	2
Yes	8
2-year overall survival rate, %	61
Neoadjuvant radiation of primary (n = 10)	
No	6
Yes	4
Relapse	
RNOH	7/10
MDACC	1/13

Clinical data are only available for 23/25 of the patients collected.

### PRMS resembles other complex sarcomas in genomic complexity

**Figure 1:**
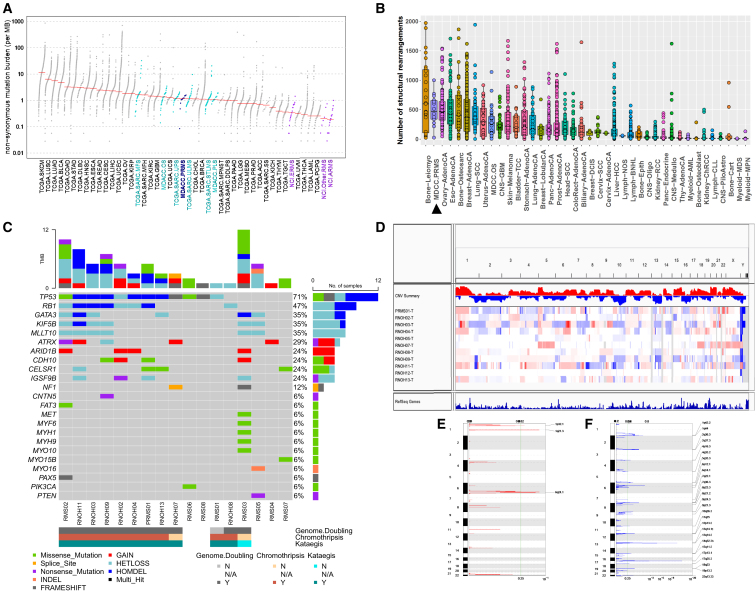
Genomic features of PRMS Whole genomes of PRMS samples were taken for somatic point mutation calling and copy-number alteration assessments. (A and B) Non-synonymous mutation burden (A) and number of rearrangements (B) as compared to cancers from ICGC, our in-house MDACC sarcomas, and ARMS and ERMS from St. Jude Children’s Research Hospital. (C) Mutation landscape including point mutations and copy-number alterations of the most frequently mutated genes based on genome and exome sequencing, including presence of chromothripsis and kataegis. As a note, one patient did not have any mutations in the genes shown. (D) Genome-wide copy-number profiles. (E) GISTIC peaks for copy-number gains. (F) GISTIC peaks for copy-number losses. RNOH, Royal National Orthopedic Hospital; MDACC, UT MD Anderson Cancer Center; CC, Cleveland Clinic; NCI, National Cancer Institute; INDEL, insertion/deletion; HETLOSS, heterozygous copy-number loss; HOMODEL, homozygous deletion; Multi_Hit, two changes, presumably on both chromosomes.

Whole-genome profiling revealed that the non-synonymous point mutation burden (median = 39 per MB) and the median number of rearrangements per patient (median = 474) were similar in PRMS, undifferentiated pleomorphic sarcoma (UPS), and osteosarcoma (Figures 1A and 1B). PRMS had a significantly higher median point mutation burden than ARMS (p = 8.36e−06) and ERMS (p = 2.58e−05) (Figure 1A), with C>T and T>C as the most frequent base pair changes (Figure S2). As with osteosarcoma, few of the neoantigen point mutations that were called in PRMS were also detected in the RNA-seq data, suggesting that many are not expressed (Figure S3).^20^

The most common deleterious somatic mutations in PRMS were those in *TP53* and *RB1*, located within 17p and 13q, respectively (Figure 1C). These regions were the most frequently deleted across patients, similar to ARMS and ERMS (Figures 1D and 1F).^5^^,^^39^ Breakpoints within intron 1 accounted for two of the *TP53* rearrangements, which is not significantly different than what we previously reported in osteosarcoma (8/35, chi-squared statistic 0.14, p = 0.71).^20^ The other breakpoints were located in intron 10 (n = 1) and exon 1 (n = 3) of *TP53*. No germline mutations in *TP53* or *RB1* were seen.

In addition, the alterations in *TP53* and *RB1* were associated with high frequencies of whole-genome doubling (11/14, 79%), kataegis (11/14, 79%), and chromothripsis (10/14, 71%) (Figure 1C). This high degree of genomic instability (genome doubling, chromothripsis, kataegis) was similar to what has been observed in UPS and osteosarcoma.^20^^,^^40^ Other recurrently mutated genes were losses of 10p (*GATA3*, *KIF5B*, *MLLT10*) and those involved in homophilic adhesion (*CDH10*, *CELSR1*, *IGSF9B*) (Figure 1C). The most prominent gains occurred on chromosome 6, where *ARID1B* resides (n = 4) (Figure 1E). Mutations in muscle-related genes were infrequent in PRMS, with only one patient sample (RMS01) having a deletion in *PAX3* and another (RMS02) having a single frameshift p.G343fs in *PAX5*. Unlike fusion-negative ARMS and ERMS that have mutations in *NRAS*, no *RAS* family mutations were detected in our samples.^41^ *NF1* is a commonly mutated gene in ARMS and ERMS,^41^ but in our PRMS cohort, we found only one patient with a splice site mutation in *NF1*.

**Figure 2:**
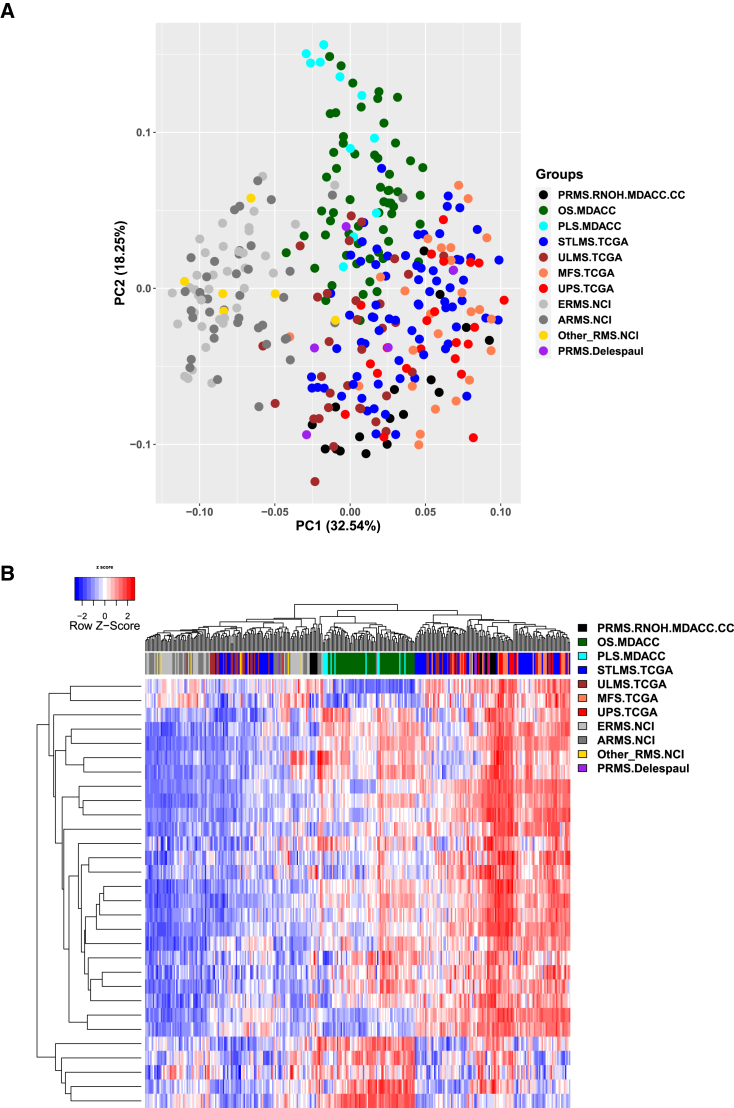
Relatedness of PRMS transcriptomes to other sarcomas The datasets here were taken from our PRMS cohort (PRMS.RNOH.MDACC.CC), TCGA, our in-house MDACC sarcomas, and ARMS and ERMS from National Cancer Institute (NCI) and the Delespaul PRMS cohort. RNA-seq normalized gene expression data compared using (A) principal-component analysis using all genes and (B) unsupervised hierarchical clustering. For each of the top 1,500 most variable genes, the normalized gene counts for all samples formed the population from which z-scores were derived. The z-scores were then combined to generate the heatmap. The cancer type abbreviations are as follows: OS, osteosarcoma; PLS, pleomorphic liposarcoma; STLMS, soft-tissue leiomyosarcoma; ULS, uterine leiomyosarcoma; MFS, myxofibrosarcoma; UPS, undifferentiated pleomorphic rhabdomyosarcoma; ERMS, embryonal rhabdomyosarcoma; ARMS, alveolar rhabdomyosarcoma; Other_RMS, other non- ERMS and non-ARMS rhabdomyosarcomas.

**Figure 3:**
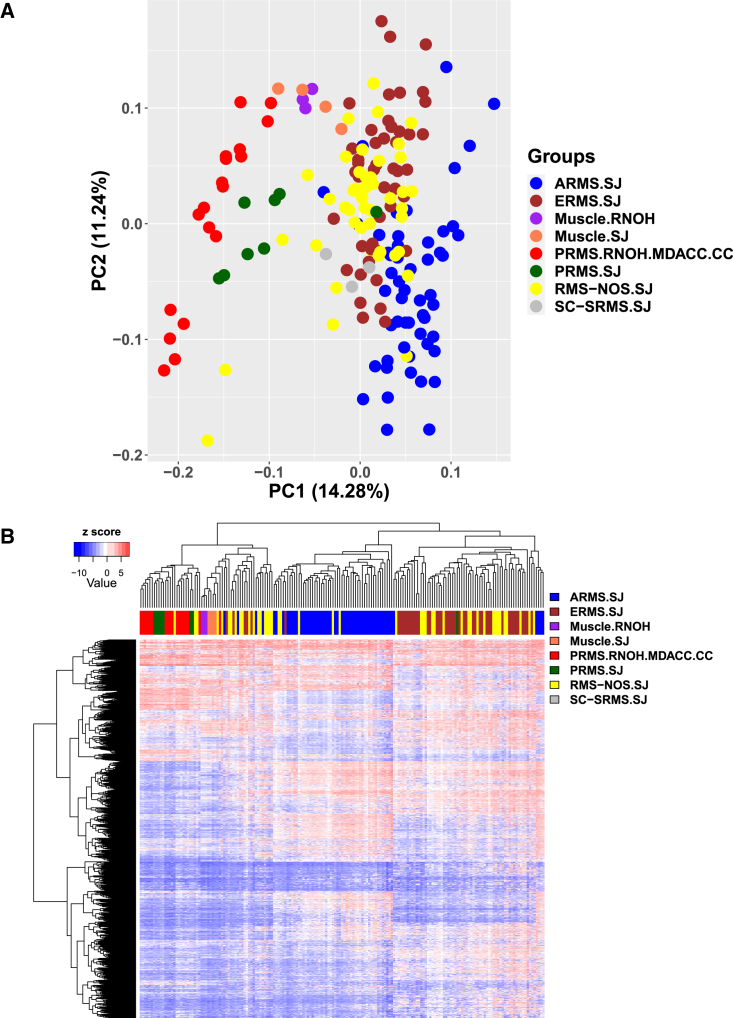
Relatedness of PRMS methylomes to other rhabdomyosarcomas and adjacent muscle Illumina EPIC data from our cohort and other public datasets were compared using (A) principal-component analysis using all probes and (B) unsupervised hierarchical clustering based on the top 10,000 most variable probes. For each probe, the z-score was calculated across samples based on the M-values. The z-scores were used together to generate the heatmap. New abbreviations for this figure: SC-SRMS, spindle cell/sclerosing rhabdomyosarcoma; SJ, St. Jude Children’s Hospital.

To improve our understanding of PRMS, we compared the transcriptomes between our cohort and multiple sarcomas of other histologic subtypes, as well as an independent PRMS cohort from Delespaul et al. (GEO: GSE75885)^42^ (see materials and methods). Unsupervised clustering of these ssGSEA scores revealed that PRMS samples were more similar to UPS, leiomyosarcoma, and myxofibrosarcoma than to osteosarcoma and pleomorphic liposarcoma and were markedly different from ARMS and ERMS (Figure 2). Similarly, comparison of methylation data from our PRMS cohort and multiple rhabdomyosarcoma subtypes from the St. Jude Children’s Research Hospital cohort^5^ as well as adjacent normal muscle tissue showed that PRMS methylomes were more highly related to those of normal skeletal muscle than to those of ARMS and ERMS (Figure 3).

### PAX3 and DMD are associated with age at diagnosis

**Figure 4:**
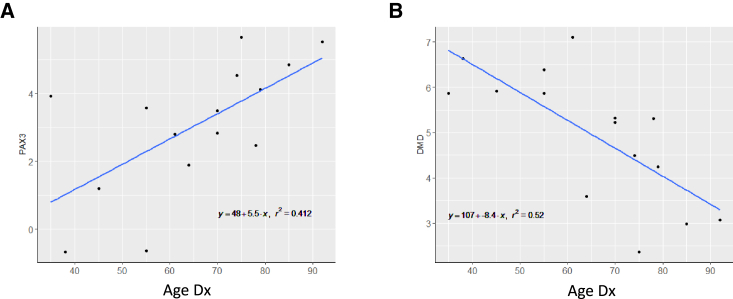
*PAX3* and *DMD* are associated with age at diagnosis (A) *PAX3* gene expression levels are positively correlated with age at diagnosis of the primary PRMS. (B) *DMD* gene expression levels are negatively correlated with age at diagnosis of the primary PRMS. Linear regression equations are shown with coefficients of determination. Age Dx, age at diagnosis.

The pathological classification of PRMS is partly based on skeletal muscle features. To further characterize the possible clinical impact of these features, we first selected 19 well-established markers of skeletal muscle progenitors and differentiation (Table S2). The homeobox transcription factor *PAX3* and *DMD* gene expression correlated with age at diagnosis positively (*PAX3*, r^2^ = 0.412, p = 0.00983) and negatively, (*DMD*, r^2^ = 0.52, p = 0.002422), respectively (Figure 4). These associations were not observed in three datasets of healthy skeletal muscle when using age as a continuous variable against gene expression (Pearson correlation p > 0.05) (Figures S4–S7).^43^^,^^44^

### Subgroups of skeletal muscle features in rhabdomyosarcoma

We then expanded this comparison with annotated skeletal muscle-related gene sets from MSigDB (skeletal muscle development, contraction, metabolism, and diseases; Figure S8). Pathways that were enriched in a subset of ARMS and ERMS but that were absent in PRMS were related to muscle filaments and structure (Figure S8A). Several ERMS samples clustered with PRMS samples according to their high scores in pathways related to muscle hypertrophy and atrophy (Figure S8C). Interestingly, two subgroups of PRMS emerged that differed in several pathways, including girdle muscle weakness (Figure S8D). When the patients with PRMS were separated into two groups according to the median scores in hip, shoulder, and limb girdle muscle weakness, those patients with a high score had worse OS than those with a low score (Figures S8E–S8G). Lower limb girdle muscle weakness scores were associated with high immune infiltrate level, discussed below (Figures S8H–S8J).

### PRMS immune infiltrate levels align with those of complex sarcomas that respond to immune checkpoint blockade

**Figure 5:**
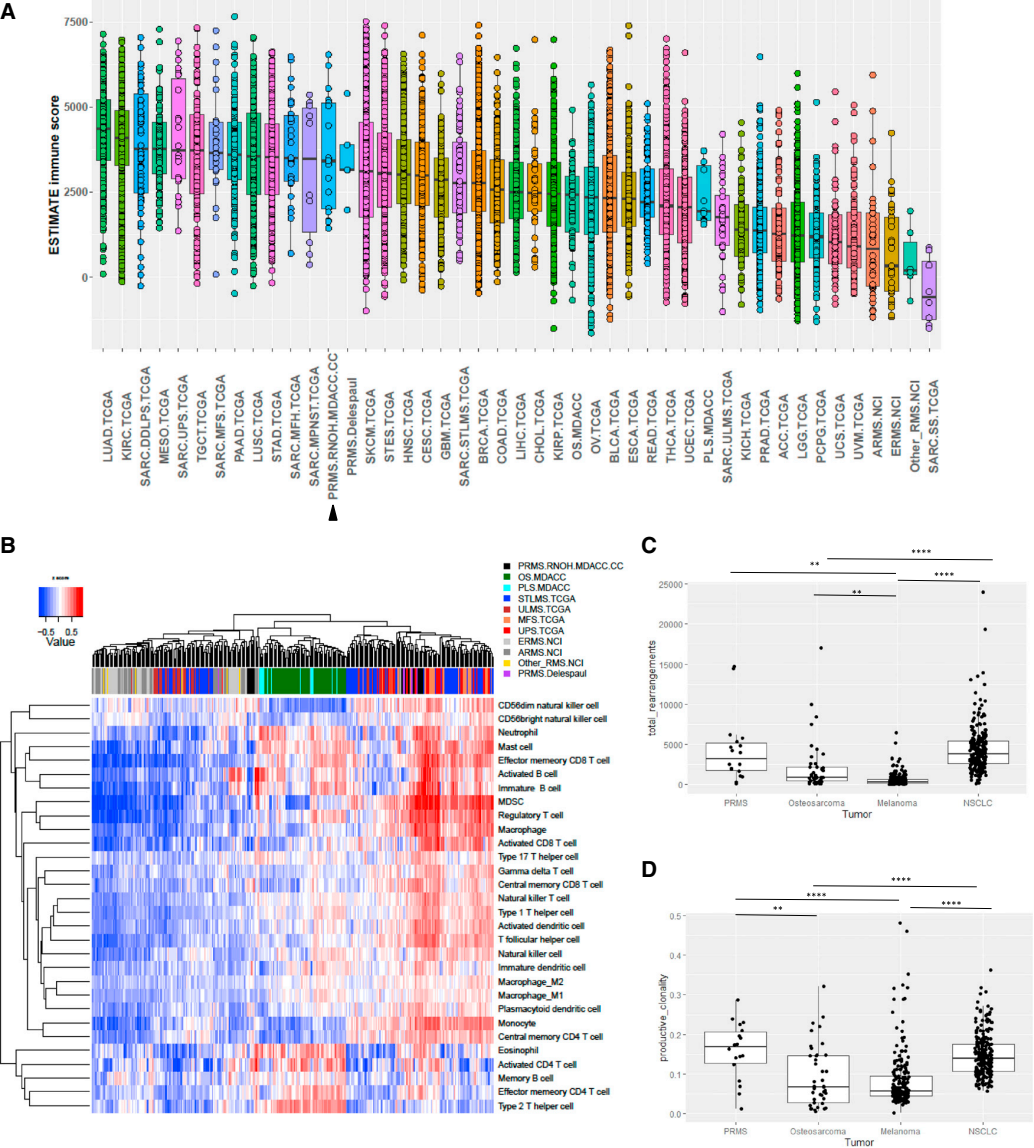
Immune profile of PRMS (A and B) ESTIMATE immune score.^31^ (A) Boxplots of the ESTIMATE immune scores (B) Immune cell type gene set scores based on Charoentong et al.^46^ were derived for each sample. Then, the z-scores for each cell type were calculated across samples and used in unsupervised hierarchical clustering. (C and D) T cell receptor sequencing in PRMS as compared with osteosarcoma,^20^ melanoma^33^, and NSCLC^34^, shown as boxplots for the total number of receptor rearrangements (C) and productive clonality (D). ∗ p < 0.05; **∗∗** p < 0.01; ∗∗∗ p < 0.001**.**

Given that the mutation profiles of PRMS were similar to those of complex sarcoma subtypes that respond to immune checkpoint blockade (ICB) such as UPS,^45^ we examined whether the immune landscapes of PRMS and these types of sarcomas were also similar (Figure 5A). The median ESTIMATE^31^ immune scores for the Delespaul PRMS cohort (PRMS.Delespaul) and our PRMS cohort (PRMS.RNOH.MDACC.CC) were similar. These PRMS median scores were slightly lower than those for other complex sarcomas, including dedifferentiated liposarcoma (SARC.DDLPS.TCGA, p = 0.313) and UPS (SARC.UPS.TCGA, p = 0.173) and were similar to lung squamous cell carcinoma (LUSC.TCGA, p = 0.539) and skin cutaneous melanoma (SKCM.TCGA, p = 0.838). All of these tumor types have exhibited responses to ICB. In contrast, the median ESTIMATE scores for PRMS were significantly higher than those of ERMS (ERMS.NCI, p = 2.99e−07) and of fusion-driven sarcomas that have lower mutational burden and do not respond well to ICB such as ARMS (ARMS.NCI, p = 1.05e−07) and synovial sarcoma (SARC.SS.TCGA, p = 9.25e−07).

To gain further insights into the composition of the infiltrating immune cells in our PRMS samples and those in the Delespaul PRMS cohort (PRMS.Delespaul), we applied ssGSEA to characterize these and other sarcoma subtypes^46^ (Figure 5B). Up to half of the PRMS samples belonged to the cluster with a high level of immune infiltrate, which is enriched with most types of immune cells. In contrast, the majority of the ARMS and ERMS samples belonged to the cluster with lower immune infiltrate. The most prominent upregulated genes in PRMS as compared with ARMS and ERMS were related to mutation burden and antigen presentation (Figure 1A; Tables S3 and S4), suggesting that genomic alterations leading to differences in antigen presentation may be key etiological factors in PRMS.

Further characterization of the T cells and B cells were performed. The total numbers of TCR rearrangements and productive clonality in our PRMS specimens were similar to those found in non-small cell lung carcinoma and were higher than in melanoma and osteosarcoma (Figures 5C and 5D). These results suggest that there is substantial T cell activation in PRMS. More than half of our samples (9/14, 64%) were positive for tertiary lymphoid structures by CD20 immunoreactivity of B cells (Table S5). B-cells were indicative for response to ICB^47^ and promising outcomes after ICB were recently observed in two cases of PRMS.^15^^,^^48^

### Higher immune infiltrate is associated with improved OS in PRMS

**Figure 6:**
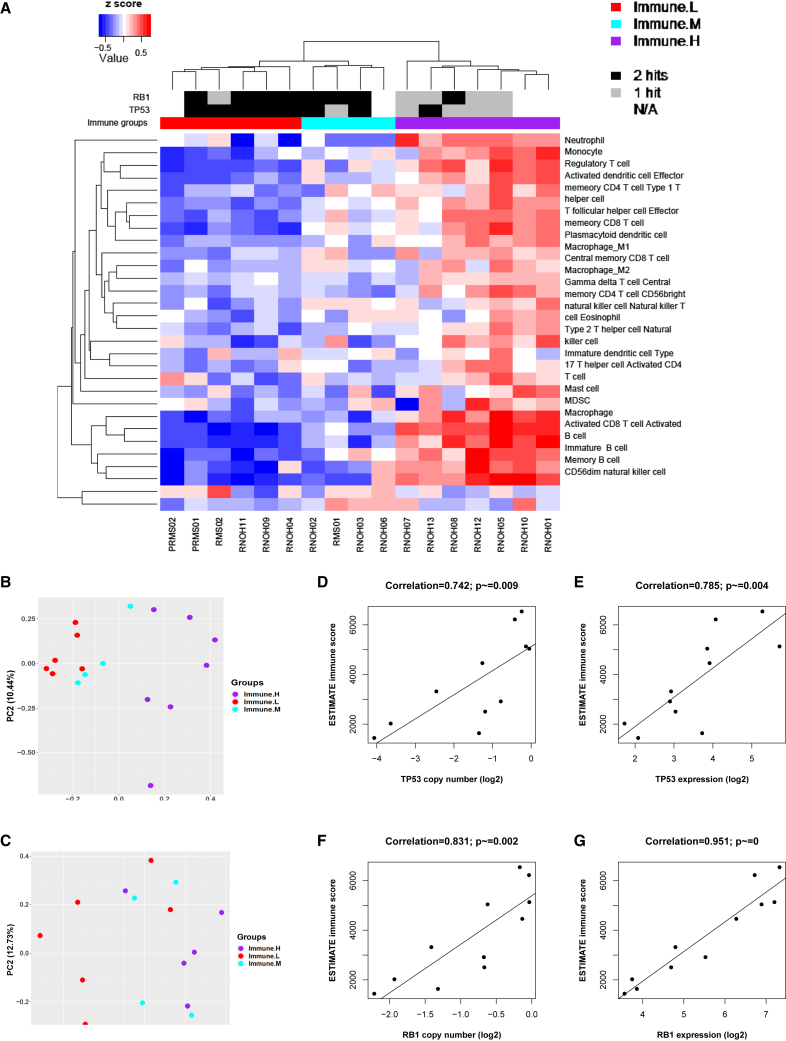
Immune infiltrate levels (A) Hierarchical clustering of immune cell types across PRMS samples from RNOH and MDACC (US) as well as adjacent normal skeletal muscle (normal). Gene sets based on Charoentong et al.^28^ (B and C) Principal-component analyses based on transcriptomes (B) and methylomes (C) of PRMS and colored according to immune infiltrate level. (D–G) Pearson correlation analysis between *TP53* copy-number log2 scores (D), *TP53* normalized gene counts (E), *RB1* copy number log2 scores (F), and *RB1* normalized gene counts (G) and ESTIMATE immune scores. Correlation coefficients and p values are shown.

To examine the heterogeneity of the immune infiltrate in our PRMS samples more closely, immune cell-type deconvolution by ssGSEA was used for focused analysis.^46^ Hierarchical clustering of the immune cell landscape revealed three distinct clusters that indicated low, intermediate, and high levels of immune infiltrate, respectively (Figure 6A). These clusters also corresponded significantly with other immune infiltrate scores (ESTIMATE and TIMER; Figure S9) and with the immune staining of T cell markers (Figure S9). These differing levels of immune infiltrate were among the major factors distinguishing subgroups of patients with PRMS when examining the cohort at both the gene expression and methylation levels (Figures 6B and 6C). Out of the clinical variables tested (age, tumor size, tumor site, and survival), patients with high immune infiltrates had significantly better outcomes (Figure S10). In a corollary Cox regression analysis, OS was better in patients with higher ESTIMATE scores (p = 0.007).

### Potential immune-modulatory mechanisms in PRMS

Several potential immune-modulatory mechanisms were associated with immune infiltrate in our PRMS samples. High burden of copy-number loss and global methylation levels at LINE-1 CpG sites were significantly correlated with high levels of immune infiltrate (Table S6). Since methylation is associated with copy number,^49^ multiple linear regression was used to compare several genomic/epigenomic factors and immune score. The burden of copy-number losses had the strongest association with immune score (p = 0.04). We further applied an integration analysis to identify genes with copy-number alterations that were significantly associated with immune infiltrate and found that copy-number loss and gene expression of *TP53* and *RB1* were both significantly negatively correlated with ESTIMATE scores (Figures 6A and 6E–6H). In addition, samples with low immune infiltrate levels were more likely to have homozygous alterations in both *TP53* and *RB1* than those with high immune infiltrate levels (Fisher’s exact test, p = 0.048). This finding was recapitulated in other complex sarcomas. Lower expression levels of *TP53* and *RB1* were associated with lower ESTIMATE scores for osteosarcoma, leiomyosarcoma (SARC.STLMS.TCGA), and UPS (SARC.UPS.TCGA) (Tables S7 and S8). These lower levels of *TP53* may be due to copy-number loss for osteosarcoma and leiomyosarcoma but not for UPS (Tables S7 and S8). Copy-number loss of *RB1* was associated with lower immune scores in leiomyosarcoma but not in osteosarcoma and UPS (Tables S7 and S8). Therefore, immune infiltration may be affected by the expression of these tumor suppressors.

Pathways that were enriched in our PRMS samples with high immune infiltrate levels compared with our samples with low immune infiltrate levels included cytokine-cytokine signaling, antigen presentation, JAK-STAT, and TCR pathways. These same pathways were previously identified as enriched in the high immune infiltrate group in osteosarcoma.^20^ More detailed examination of these pathways revealed that *IFNG* and the key JAK-STAT signaling members *JAK3*, *STAT1*, *STAT4*, *STAT5A*, and *STAT6* had significantly greater expression in the high immune infiltrate group (Figures S11A–S11F). *CIITA* and *B2M*, the master controllers of antigen presentation and subsequent T cell activation, were >2-fold higher in the high immune level group as compared with the low immune level group (Figures S11G and S11H). However, multiple immune-suppressive molecules were also significantly elevated in the high immune infiltrate group: *LAG3*, *IDO1*, and *IFI30* (Figures S12A–S12C). *TGFB1* inhibits CD8+ T cell differentiation into cytolytic states and was elevated in all PRMS samples compared with healthy muscle regardless of immune infiltrate (Figure S11D).^50^ Of note, *CD274* (PDL1) was not significantly different across immune groups (Figure S13).

**Figure 7:**
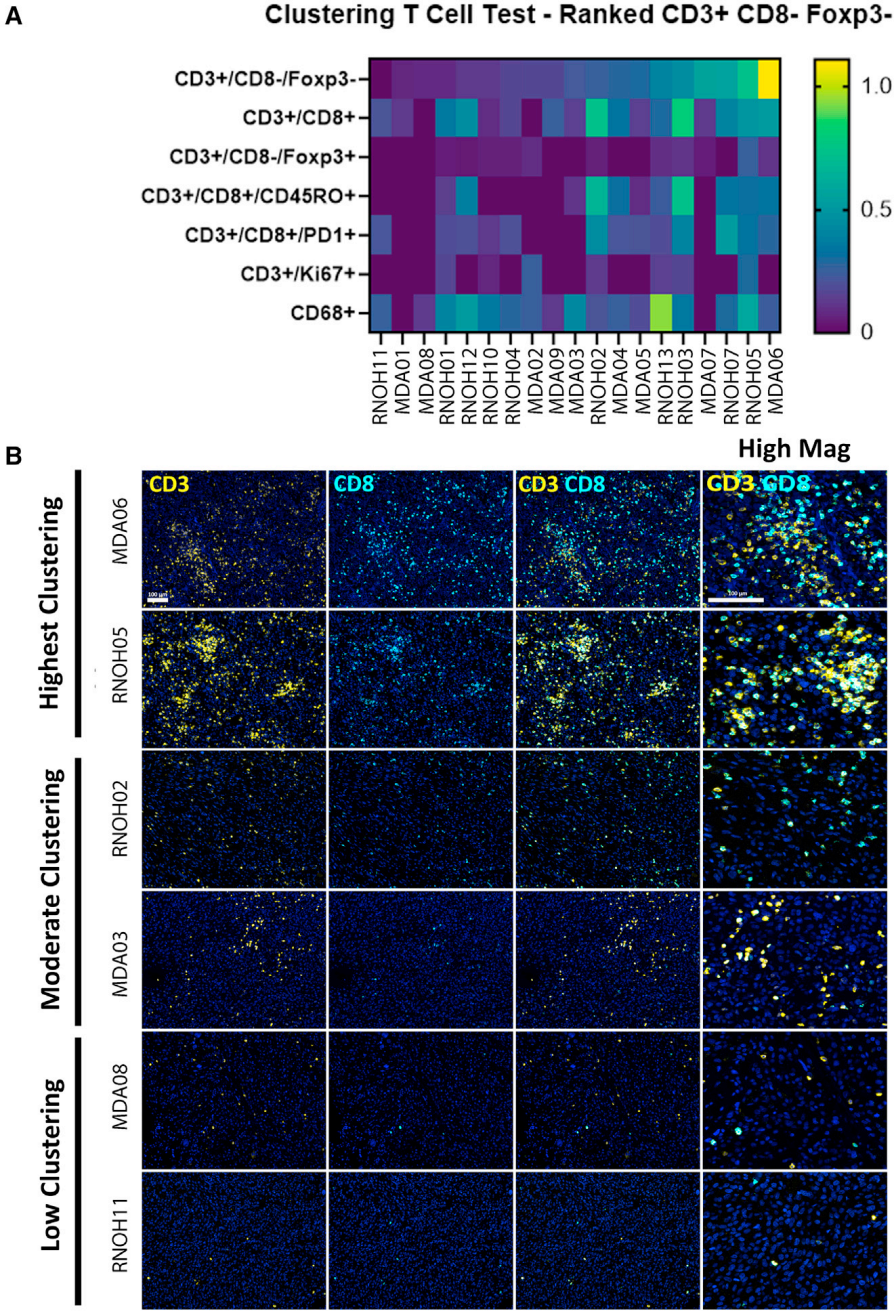
Lymphocyte aggregates in a subset of PRMS (A) Multi-immunofluorescent spatial analysis was performed to assess the physical proximities between cells according to the positivity of the 9 markers present. Here, the densities of CD3+, non-regulatory T cells (CD3+/CD8−/FOXP3−) (<15 μm) are ordered (yellow indicating high densities), compared, and contrasted with other CD3+ costains and CD68+ monocyte/macrophages monostaining. (B) Representative stains with CD3+ shown in yellow, CD8+ in light blue, and both markers with low and high magnifications. Samples with highest clustering densities reveal lymphocyte aggregates (MDA06 and RNOH05). Samples with moderate clustering do not have lymphocyte aggregates despite being positive for CD3+ and CD8+ (RHOH02 and MDA03). Finally, samples with low immune infiltrates do not have any lymphocyte aggregates (MDA08 and RNOH11).

By multiplex immunofluorescent staining of immune markers, we found two subgroups of PRMS that differed according to CD3+FOXP3− cell densities (Figure 7). One group had high aggregates of these cells, while the other had little or no staining of these cells (immune cold). These cells were not proximal to CD68+ monocyte/macrophages and were not positive for Ki67, indicating that they were not actively proliferative. The aggregates were not exclusively CD8+, indicating that they are likely mixtures of CD8+ and CD4+ T cells. When testing the density measurements against multiple immune scores and mutation aberration measurements (Table S9), these CD3+FOXP3− aggregates were negatively associated with nonsense-mediated decay scores and with *DMD* expression levels (Table S9). On the basis of these observations, we hypothesize that a subset of PRMS has lymphoid aggregates that are not actively expanding and that are related to the muscle microenvironment when there are low levels of dystrophin.

## Discussion

The lack of molecular characterizations of PRMS has impeded the development of disease-specific treatment. Using extensive multiplatform profiling, we confirmed that PRMS is highly rearranged with multiple copy-number gains and losses, and a large majority of samples harbor losses and rearrangements in *TP53* and *RB1*. These features in PRMS are similar to those of other complex sarcoma subtypes such as UPS, LMS, and OS and are in contrast to the simple genomes found in ARMS and ERMS.^51^ These similarities between PRMS and other sarcoma subtypes were further substantiated by expression and methylation profiles, which showed PRMS samples clustering more closely with complex karyotype sarcomas than with ARMS or ERMS.

In regard to their muscle-like features, levels of *DMD* in PRMS were associated with the age at diagnosis, which was not observed in the normal aging of skeletal muscle. Reduced expression of *DMD* was associated with significantly higher levels of lymphoid aggregates composed of non-regulatory CD3+ T cells.

The feature that appeared to best distinguish major subgroups of PRMS was levels of immune infiltrate. Patients with higher immune infiltrate levels may have improved OS (Figure S12), which has been observed in other cancers, including sarcomas (reviewed in Barnes and Amir^52^ and Chen et al.^53^). Lower immune infiltrate levels were associated with complete loss of both *TP53* and *RB1*. Loss of *TP53* may decrease major histocompatibility complex (MHC) class I expression, which would reduce the recruitment of immune cells such as natural killer (NK) cells and the capacity for TP53-dependent apoptosis.^54^^,^^55^ *RB1* deficiency leads to lower expression of immune cell surface receptors, complement components, and cytokines.^55^ Skeletal muscle is normally an immune-privileged site and does not express MHC class I antigen-presenting genes.^56^ In addition, the aging process in skeletal muscle creates an immunosuppressive environment in otherwise healthy individuals.^43^ Therefore, it was surprising to see that the overall levels of T cells in PRMS were higher than or equivalent to those found in non-small lung cancer and melanoma, for which mutation burden is high and immune checkpoint inhibitors have shown success. However, the T cell activity in PRMS is potentially hampered by immunosuppressive mechanisms such as transforming growth factor β (TGF-β). Given our small cohort, this will require further investigation in additional samples.

Together, the levels of immune infiltrate and tumor mutation burden and the presence of expanded and activated T cells in a large proportion of these tumors and in the majority of cases with tertiary lymphoid structures suggest that immune checkpoint inhibitors coupled with concomitant suppression of TGF-β signaling may be a viable option for a subgroup of patients.^45^^,^^47^^,^^57^ This study establishes a comparative framework for further investigation into the biology of PRMS while indicating potential therapeutic strategies.

## Data and code availability

Sequencing data are available through the European Genome-Phenome Archive: EGAS00001007230 and Adaptive immuneACCESS: 10.21417/HCB2023HGGA.

## Web resources

GraphPad, graphpad.com.

Online Catalog of Human Genes and Genetic Disorders, omim.org.

## Supplemental information

Data S1. Tables S1–S9

Document S2. Article plus supplemental information
