# Supplementary material for: Complete loss of TP53 and RB1 is associated with complex genome and low immune infiltrate in pleomorphic rhabdomyosarcoma
Source: HGG Adv. 2023 Jul 19;4(4):100224. doi: 10.1016/j.xhgg.2023.100224 (PMC10428123; doi:10.1016/j.xhgg.2023.100224)
Supplement: Document S1. Figures S1–S13 and supplemental — methods [file mmc1.pdf]

**HGGA, Volume *4***

## Supplemental information

# Complete loss of *TP53* and *RB1* is associated with complex genome and low immune infiltrate in pleomorphic rhabdomyosarcoma

**Hannah C. Beird, Chia-Chin Wu, Michael Nakazawa, Davis Ingram, Joseph R. Daniele, Rossana Lazcano, Latasha Little, Christopher Davies, Najat C. Daw, Khalida Wani, Wei-Lien Wang, Xingzhi Song, Curtis Gumbs, Jianhua Zhang, Brian Rubin, Anthony Conley, Adrienne M. Flanagan, Alexander J. Lazar, and P. Andrew Futreal**

# Supplemental Figures

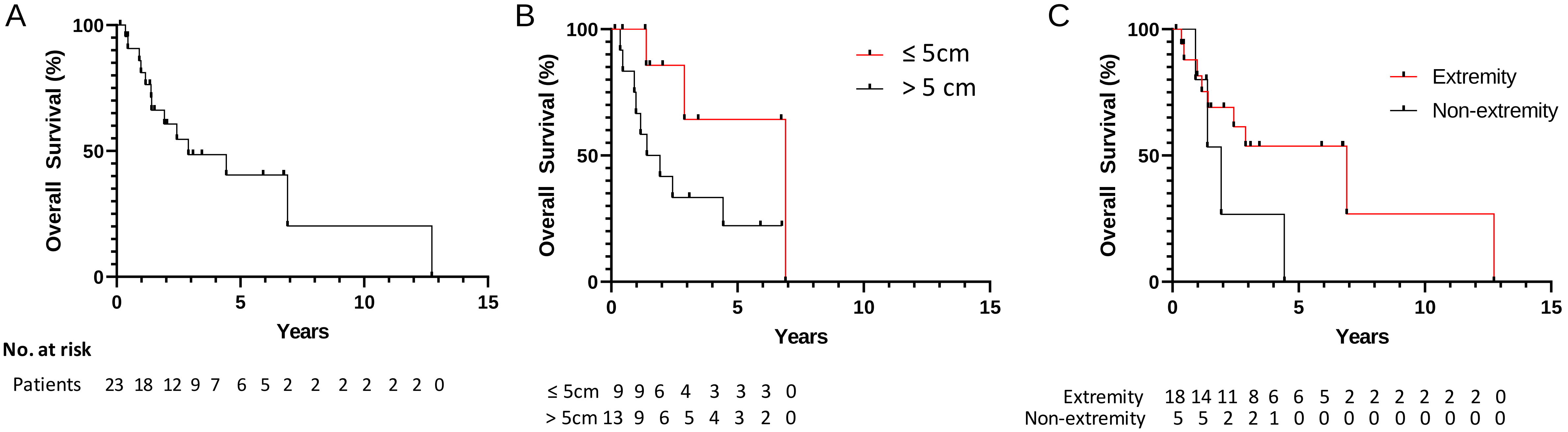


**Figure S1.** The impact of clinicopathologic variables on overall survival. Available clinical data were used in Kaplan-meier curves for 22 PRMS patients to determine overall survival of: **A.** Entire cohort **B.** Median tumor size cutoff used in rhabdomyosarcoma staging. **D.** Primary tumor site. Extremity: leg, forearm, thigh, knee, calf, hand. Non-extremity: neck, buttock, chest wall, lung, back, uterus.

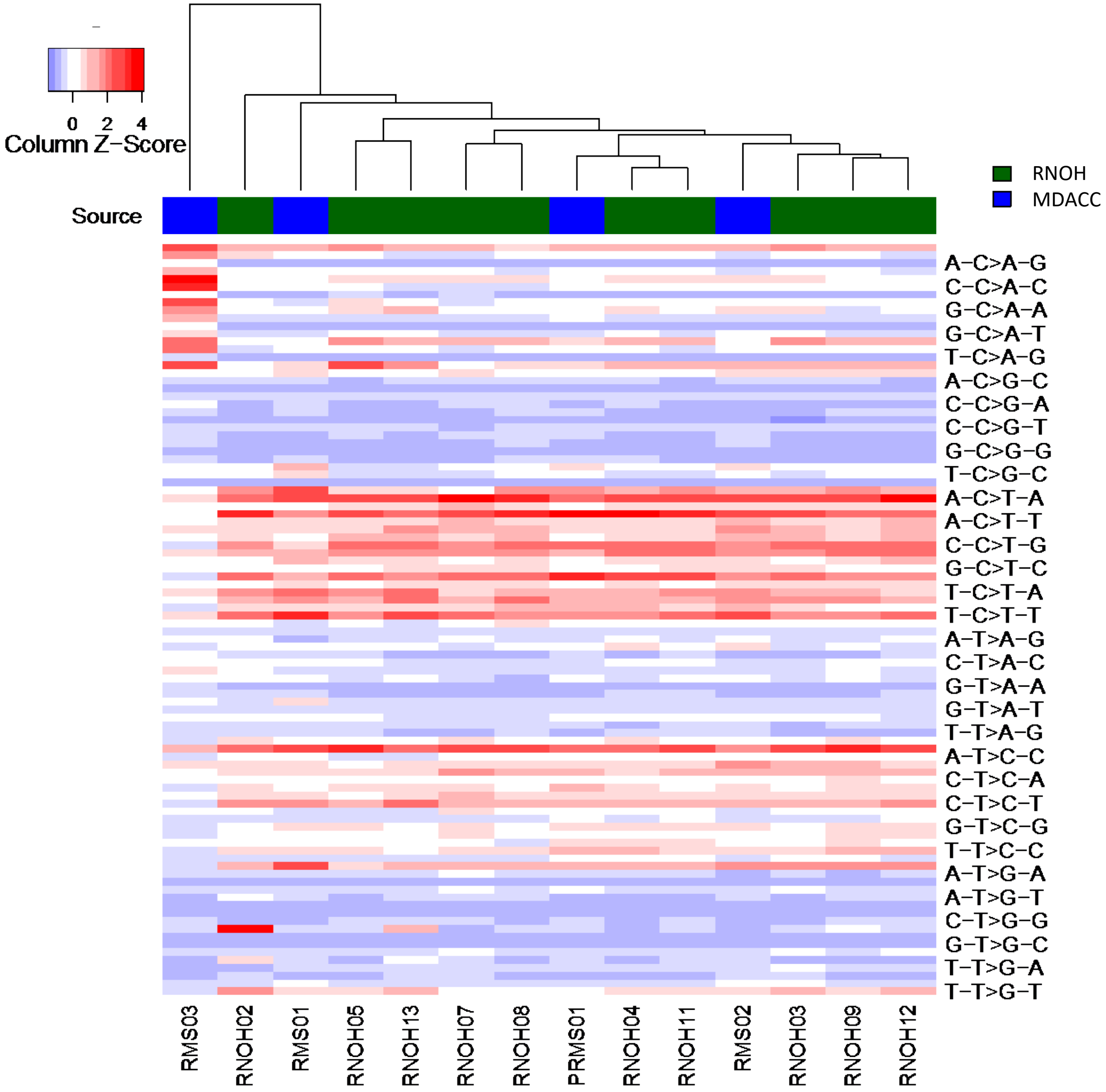


**Figure S2**: Mutation context landscape in the PRMS genomes. The number of basepair changes were normalized across each patient and then subjected to hierarchical clustering. The institution from which the samples came from are indicated: RNOH = Royal National Orthopaedic Hospical; MDACC: MD Anderson Cancer Center.

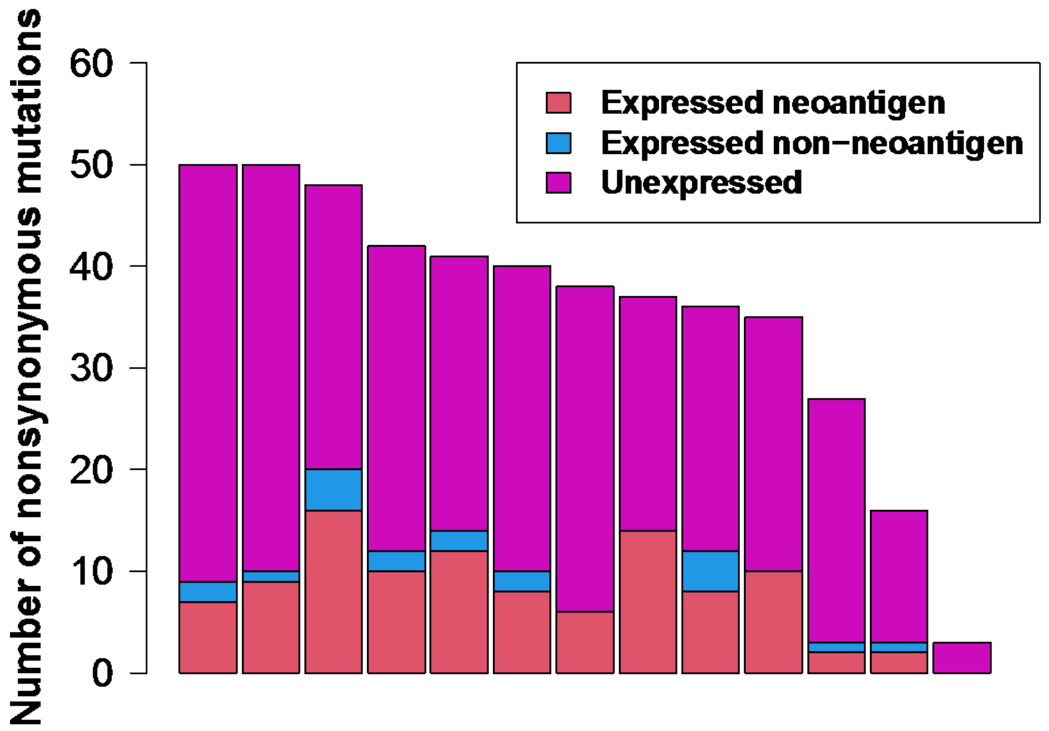


**Figure S3**: Few mutations are expressed in PRMS. Nonsynonymous mutations from whole genomes were assessed in the matching transcriptomes for expression and tallied. See Methods and Supplemental Methods for neoantigen calling.

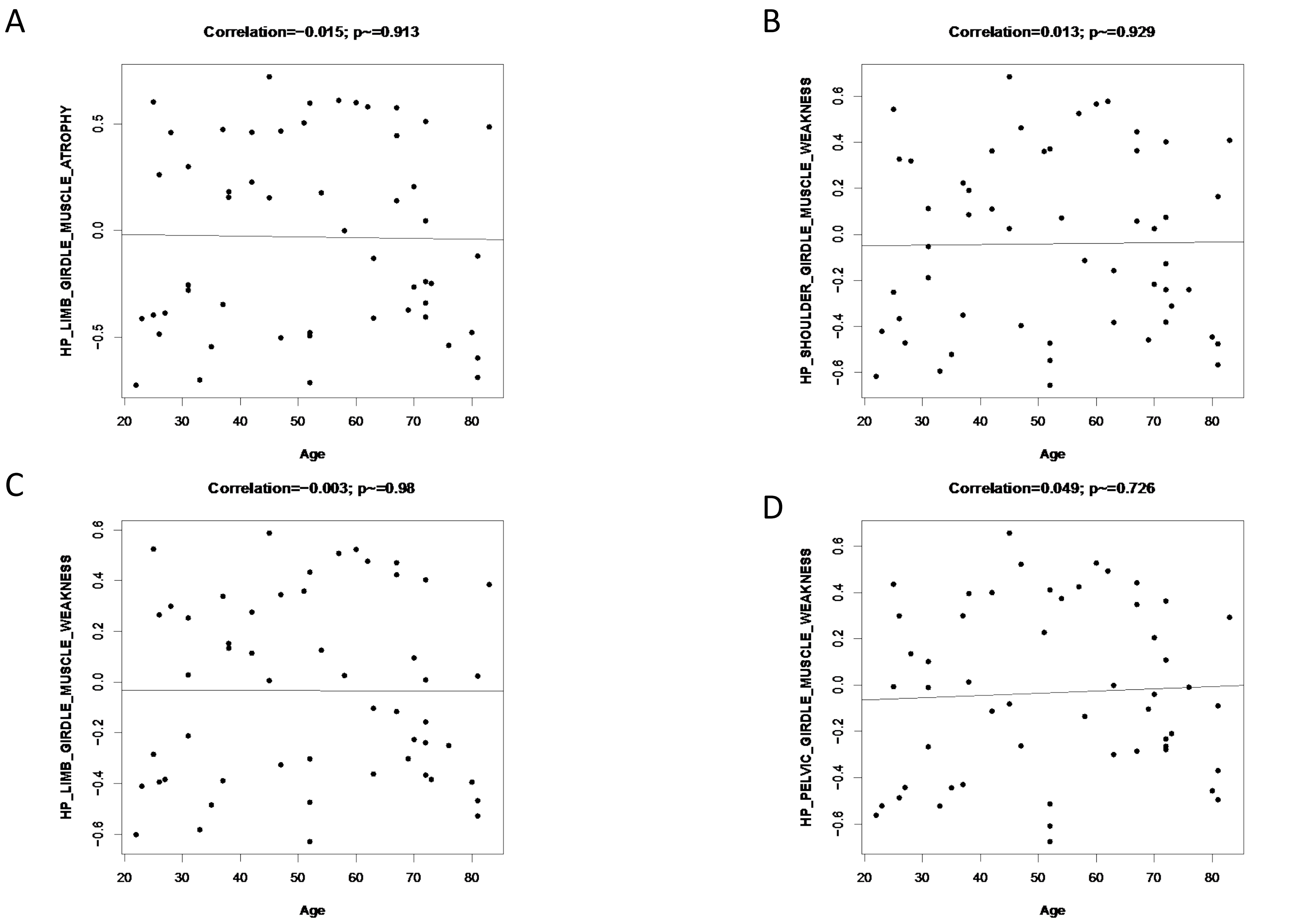


**Figure S4:** Dataset GSE164471 shows no correlation between age at diagnosis (x-axis) and the girdle muscle weakness pathway scores (y-axis). The Pearson correlation coefficients and p-values are indicated above each plot.

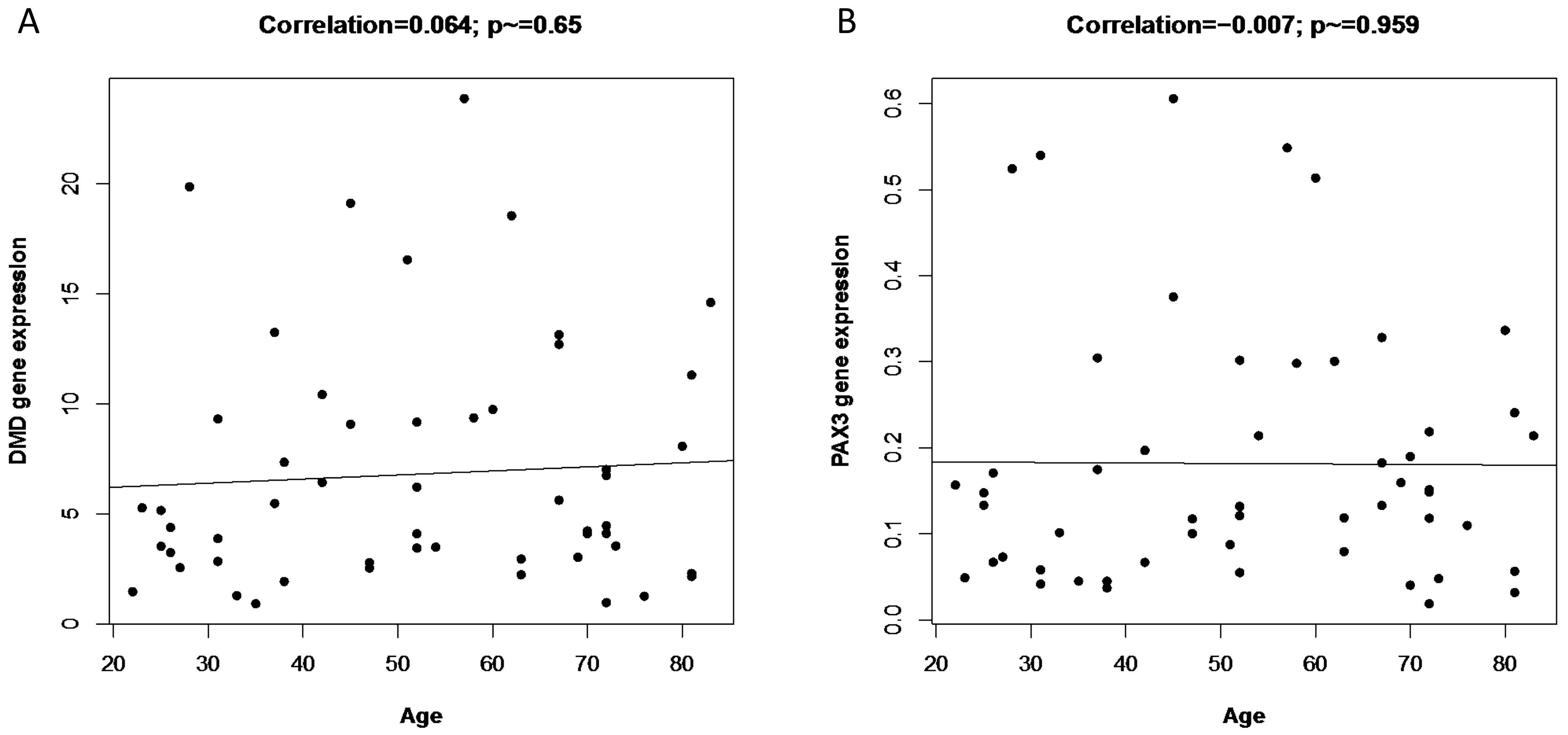


**Figure S5:** Dataset GSE164471 shows no correlation between age (x-axis) and *PAX3* & *DMD* gene expression (y-axes). The Pearson correlation coefficients and p-values are indicated above each plot.

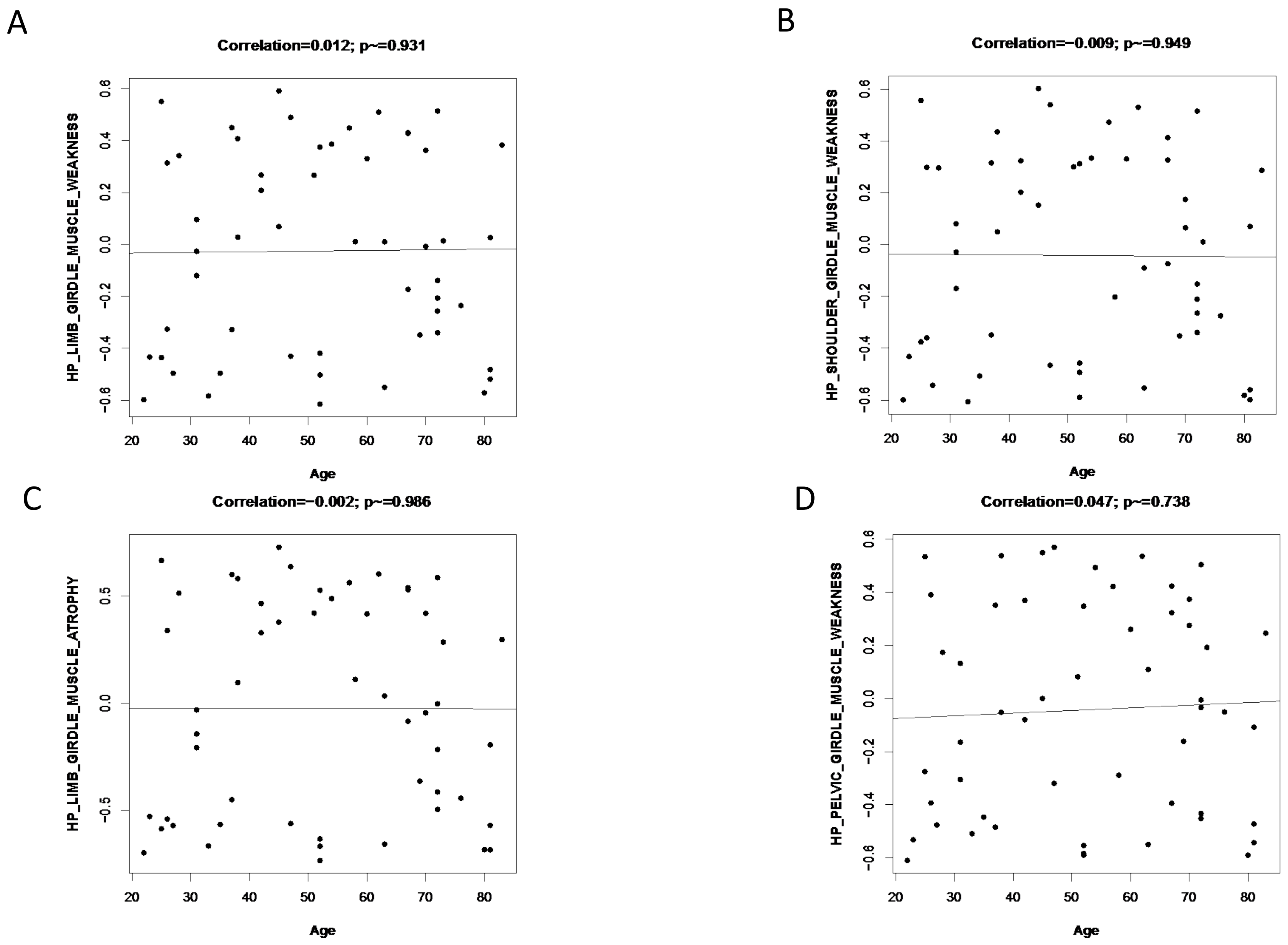


**Figure S6:** Dataset GSE129643 shows no correlation between age and the girdle muscle weakness pathway scores. The Pearson correlation coefficients and p-values are indicated above each plot.

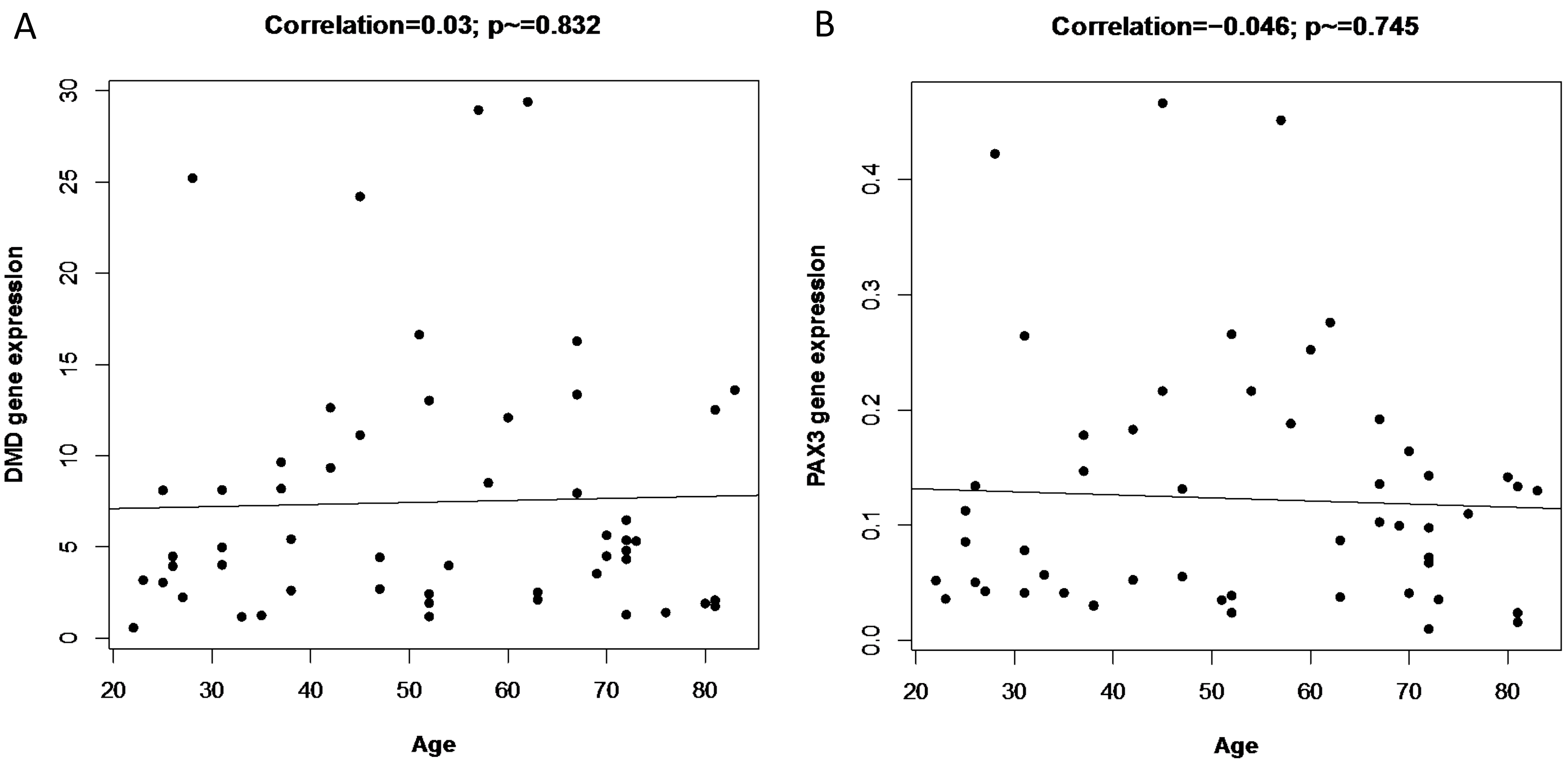


**Figure S7:** Dataset GSE129643 shows no correlation between age and *PAX3* and *DMD* gene expression. The Pearson correlation coefficients and p-values are indicated above each plot.

A

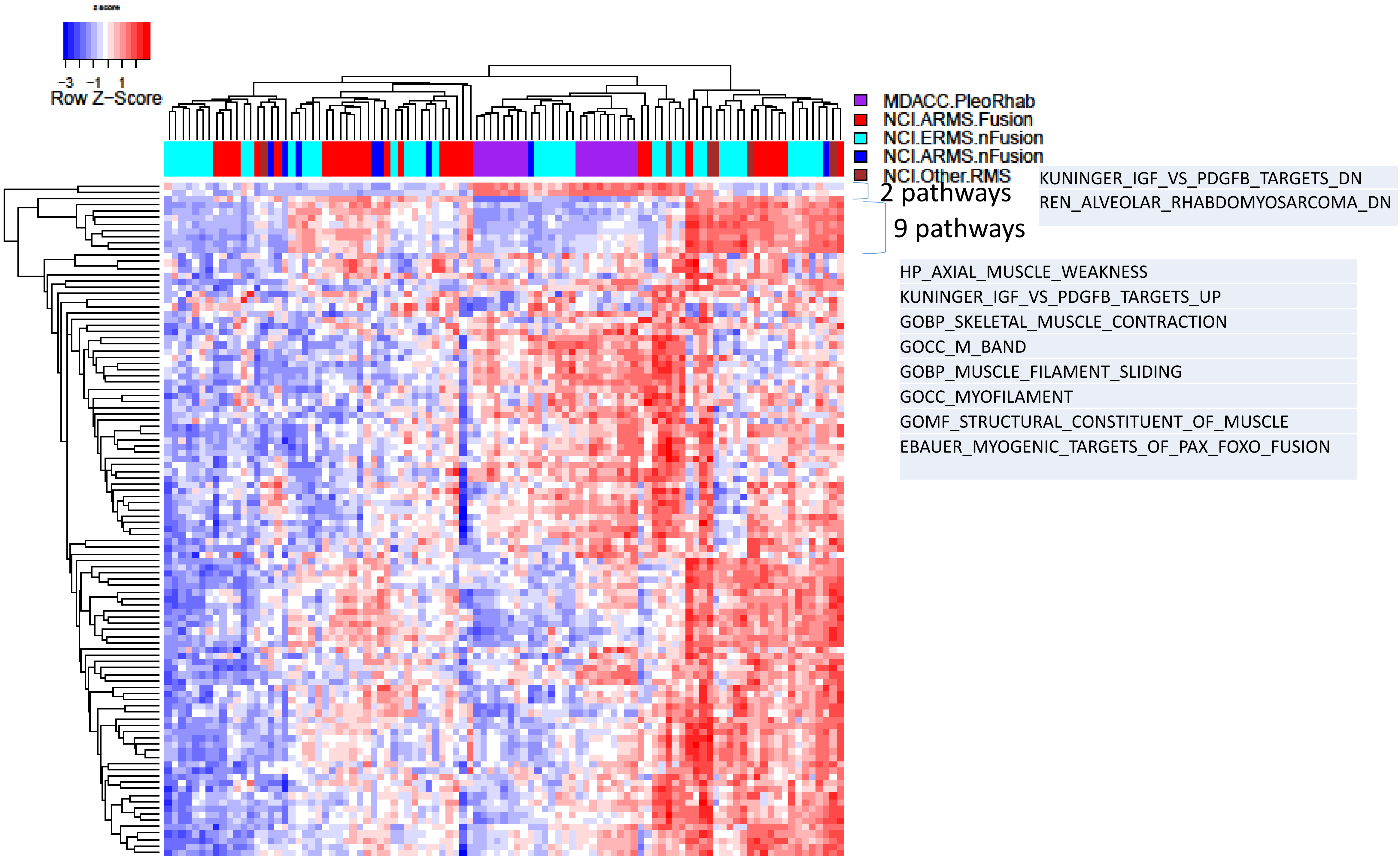
-3 -1 1
Row Z-Score
MDACC.PleoRhab
NCI.ARMS.Fusion
NCI.ERMS.nFusion
NCI.ARMS.nFusion
NCI.Other.RMS
2 pathways
9 pathways
KUNINGER_IGF_VS_PDGFB_TARGETS_DN
REN_ALVEOLAR_RHABDOMYOSARCOMA_DN
HP_AXIAL_MUSCLE_WEAKNESS
KUNINGER_IGF_VS_PDGFB_TARGETS_UP
GOBP_SKELETAL_MUSCLE_CONTRACTION
GOCC_M_BAND
GOBP_MUSCLE_FILAMENT_SLIDING
GOCC_MYOFILAMENT
GOMF_STRUCTURAL_CONSTITUENT_OF_MUSCLE
EBAUER_MYOGENIC_TARGETS_OF_PAX_FOXO_FUSION

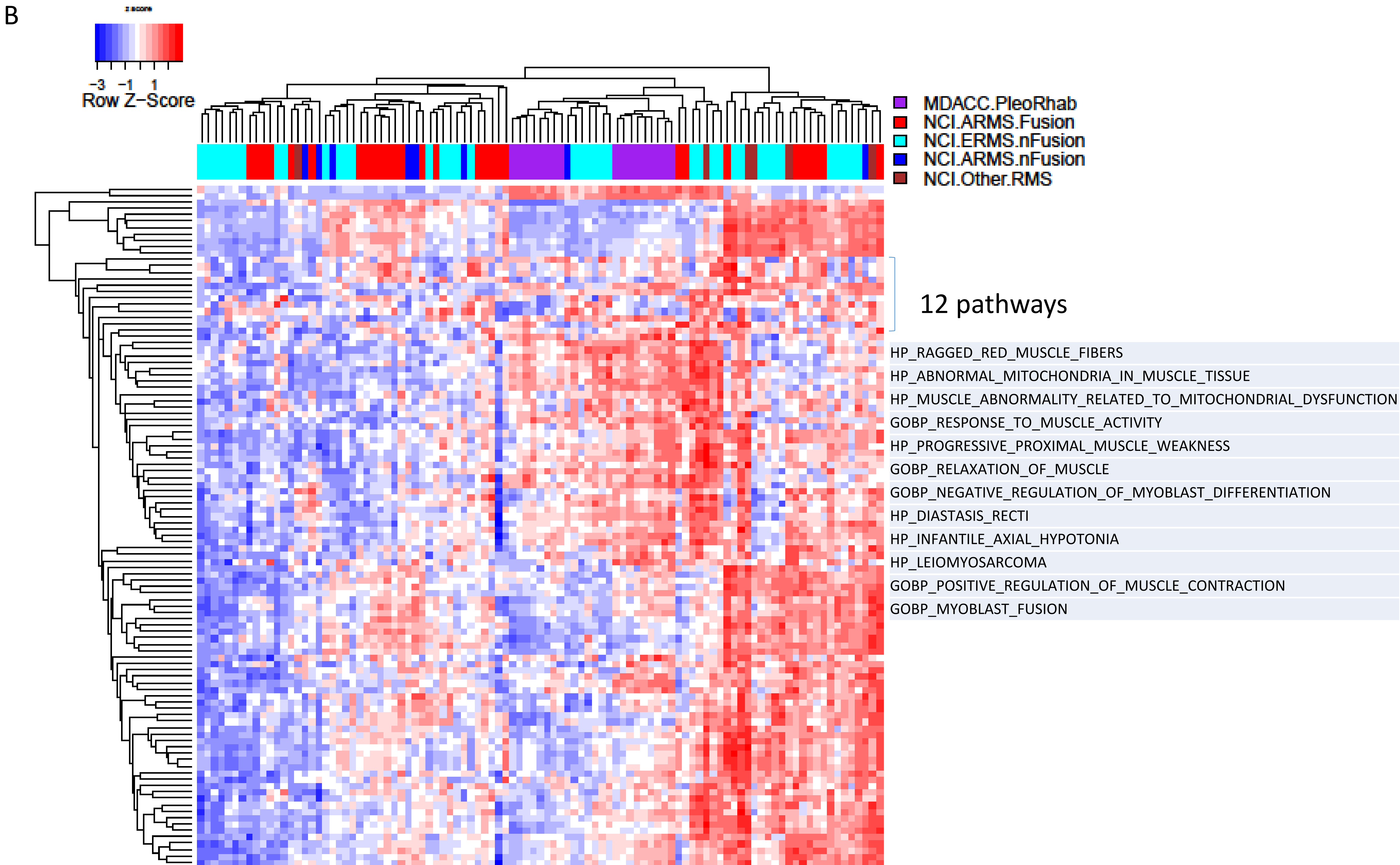
B
−3 −1 1
Row Z−Score
MDACC.PleoRhab
NCI.ARMS.Fusion
NCI.ERMS.nFusion
NCI.ARMS.nFusion
NCI.Other.RMS
12 pathways
HP_RAGGED_RED_MUSCLE_FIBERS
HP_ABNORMAL_MITOCHONDRIA_IN_MUSCLE_TISSUE
HP_MUSCLE_ABNORMALITY_RELATED_TO_MITOCHONDRIAL_DYSFUNCTION
GOBP_RESPONSE_TO_MUSCLE_ACTIVITY
HP_PROGRESSIVE_PROXIMAL_MUSCLE_WEAKNESS
GOBP_RELAXATION_OF_MUSCLE
GOBP_NEGATIVE_REGULATION_OF_MYOBLAST_DIFFERENTIATION
HP_DIASTASIS_RECTI
HP_INFANTILE_AXIAL_HYPOTONIA
HP_LEIOMYOSARCOMA
GOBP_POSITIVE_REGULATION_OF_MUSCLE_CONTRACTION
GOBP_MYOBLAST_FUSION

C

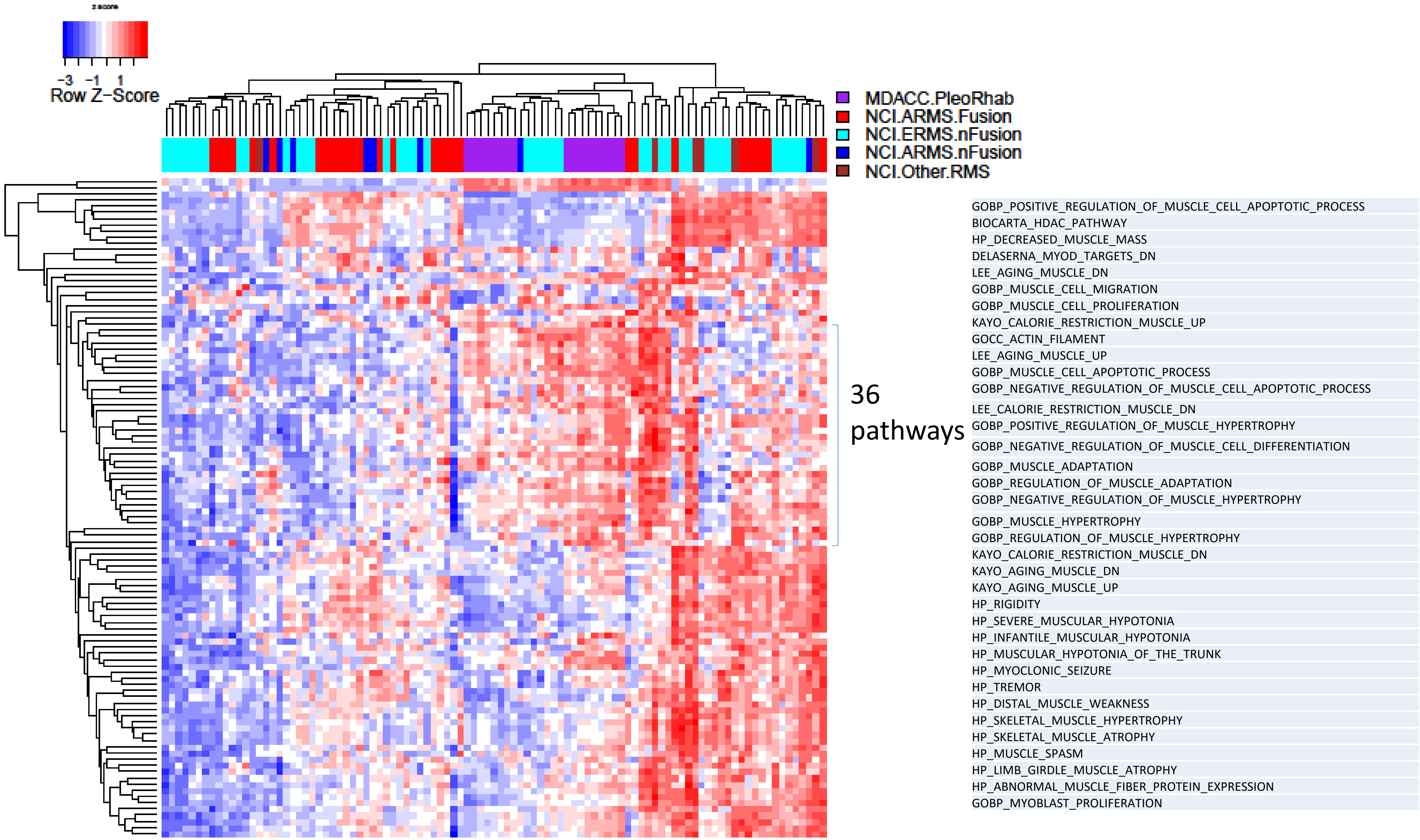

Row Z−Score
−3 −1 1
MDACC.PleoRhab
NCI.ARMS.Fusion
NCI.ERMS.nFusion
NCI.ARMS.nFusion
NCI.Other.RMS
36 pathways
GOBP_POSITIVE_REGULATION_OF_MUSCLE_CELL_APOPTOTIC_PROCESS
BIOCARTA_HDAC_PATHWAY
HP_DECREASED_MUSCLE_MASS
DELASERNA_MYOD_TARGETS_DN
LEE_AGING_MUSCLE_DN
GOBP_MUSCLE_CELL_MIGRATION
GOBP_MUSCLE_CELL_PROLIFERATION
KAYO_CALORIE_RESTRICTION_MUSCLE_UP
GOCC_ACTIN_FILAMENT
LEE_AGING_MUSCLE_UP
GOBP_MUSCLE_CELL_APOPTOTIC_PROCESS
GOBP_NEGATIVE_REGULATION_OF_MUSCLE_CELL_APOPTOTIC_PROCESS
LEE_CALORIE_RESTRICTION_MUSCLE_DN
GOBP_POSITIVE_REGULATION_OF_MUSCLE_HYPERTROPHY
GOBP_NEGATIVE_REGULATION_OF_MUSCLE_CELL_DIFFERENTIATION
GOBP_MUSCLE_ADAPTATION
GOBP_REGULATION_OF_MUSCLE_ADAPTATION
GOBP_NEGATIVE_REGULATION_OF_MUSCLE_HYPERTROPHY
GOBP_MUSCLE_HYPERTROPHY
GOBP_REGULATION_OF_MUSCLE_HYPERTROPHY
KAYO_CALORIE_RESTRICTION_MUSCLE_DN
KAYO_AGING_MUSCLE_DN
KAYO_AGING_MUSCLE_UP
HP_RIGIDITY
HP_SEVERE_MUSCULAR_HYPOTONIA
HP_INFANTILE_MUSCULAR_HYPOTONIA
HP_MUSCULAR_HYPOTONIA_OF_THE_TRUNK
HP_MYOCLONIC_SEIZURE
HP_TREMOR
HP_DISTAL_MUSCLE_WEAKNESS
HP_SKELETAL_MUSCLE_HYPERTROPHY
HP_SKELETAL_MUSCLE_ATROPHY
HP_MUSCLE_SPASM
HP_LIMB_GIRDLE_MUSCLE_ATROPHY
HP_ABNORMAL_MUSCLE_FIBER_PROTEIN_EXPRESSION
GOBP_MYOBLAST_PROLIFERATION

D

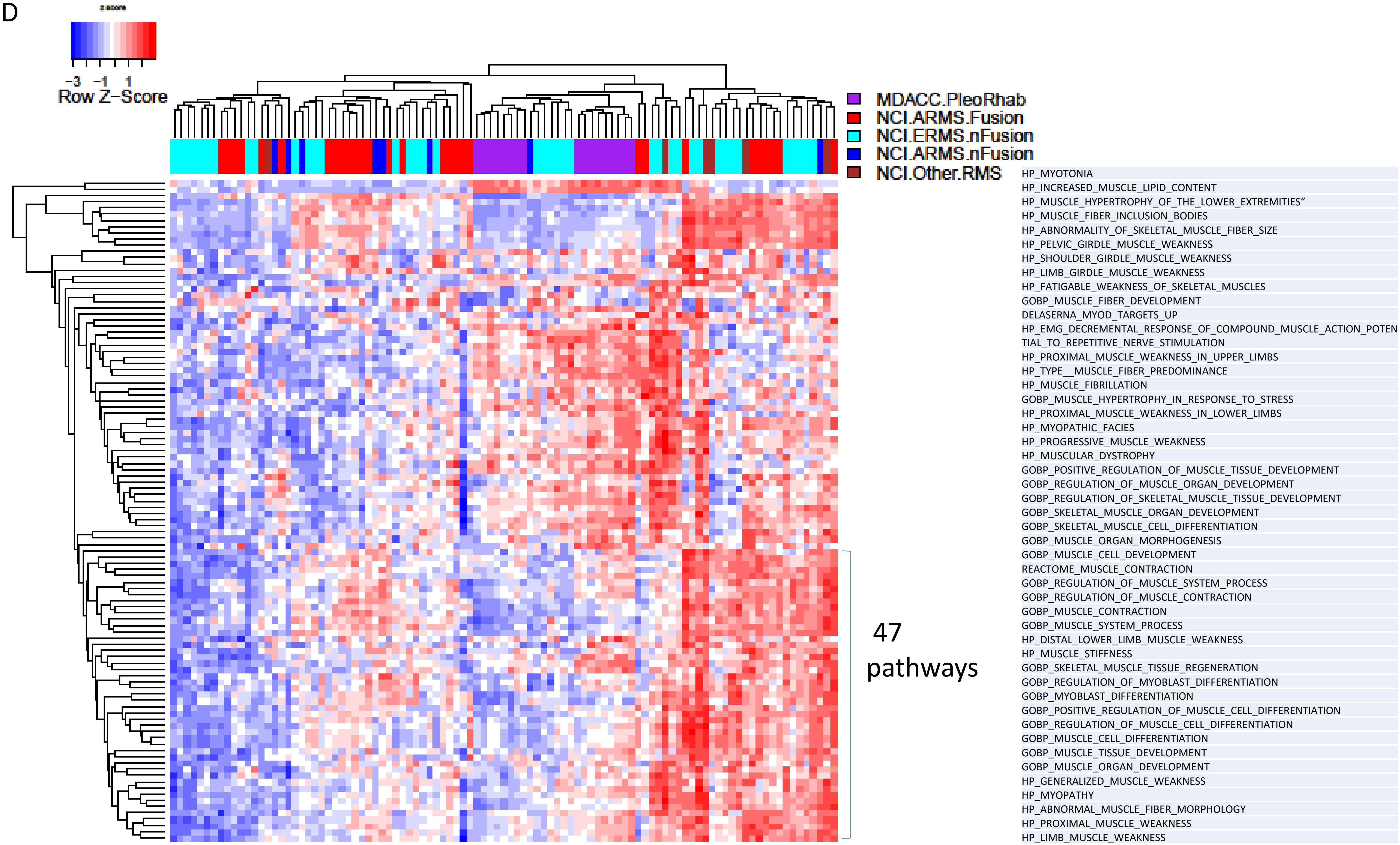
z score
-3 -1 1
Row Z-Score
MDACC.PleoRhab
NCI.ARMS.Fusion
NCI.ERMS.nFusion
NCI.ARMS.nFusion
NCI.Other.RMS
47
pathways
HP_MYOTONIA
HP_INCREASED_MUSCLE_LIPID_CONTENT
HP_MUSCLE_HYPERTROPHY_OF_THE_LOWER_EXTREMITIES“
HP_MUSCLE_FIBER_INCLUSION_BODIES
HP_ABNORMALITY_OF_SKELETAL_MUSCLE_FIBER_SIZE
HP_PELVIC_GIRDLE_MUSCLE_WEAKNESS
HP_SHOULDER_GIRDLE_MUSCLE_WEAKNESS
HP_LIMB_GIRDLE_MUSCLE_WEAKNESS
HP_FATIGABLE_WEAKNESS_OF_SKELETAL_MUSCLES
GOBP_MUSCLE_FIBER_DEVELOPMENT
DELASERNA_MYOD_TARGETS_UP
HP_EMG_DECREMENTAL_RESPONSE_OF_COMPOUND_MUSCLE_ACTION_POTEN
TIAL_TO_REPETITIVE_NERVE_STIMULATION
HP_PROXIMAL_MUSCLE_WEAKNESS_IN_UPPER_LIMBS
HP_TYPE__MUSCLE_FIBER_PREDOMINANCE
HP_MUSCLE_FIBRILLATION
GOBP_MUSCLE_HYPERTROPHY_IN_RESPONSE_TO_STRESS
HP_PROXIMAL_MUSCLE_WEAKNESS_IN_LOWER_LIMBS
HP_MYOPATHIC_FACIES
HP_PROGRESSIVE_MUSCLE_WEAKNESS
HP_MUSCULAR_DYSTROPHY
GOBP_POSITIVE_REGULATION_OF_MUSCLE_TISSUE_DEVELOPMENT
GOBP_REGULATION_OF_MUSCLE_ORGAN_DEVELOPMENT
GOBP_REGULATION_OF_SKELETAL_MUSCLE_TISSUE_DEVELOPMENT
GOBP_SKELETAL_MUSCLE_ORGAN_DEVELOPMENT
GOBP_SKELETAL_MUSCLE_CELL_DIFFERENTIATION
GOBP_MUSCLE_ORGAN_MORPHOGENESIS
GOBP_MUSCLE_CELL_DEVELOPMENT
REACTOME_MUSCLE_CONTRACTION
GOBP_REGULATION_OF_MUSCLE_SYSTEM_PROCESS
GOBP_REGULATION_OF_MUSCLE_CONTRACTION
GOBP_MUSCLE_CONTRACTION
GOBP_MUSCLE_SYSTEM_PROCESS
HP_DISTAL_LOWER_LIMB_MUSCLE_WEAKNESS
HP_MUSCLE_STIFFNESS
GOBP_SKELETAL_MUSCLE_TISSUE_REGENERATION
GOBP_REGULATION_OF_MYOBLAST_DIFFERENTIATION
GOBP_MYOBLAST_DIFFERENTIATION
GOBP_POSITIVE_REGULATION_OF_MUSCLE_CELL_DIFFERENTIATION
GOBP_REGULATION_OF_MUSCLE_CELL_DIFFERENTIATION
GOBP_MUSCLE_CELL_DIFFERENTIATION
GOBP_MUSCLE_TISSUE_DEVELOPMENT
GOBP_MUSCLE_ORGAN_DEVELOPMENT
HP_GENERALIZED_MUSCLE_WEAKNESS
HP_MYOPATHY
HP_ABNORMAL_MUSCLE_FIBER_MORPHOLOGY
HP_PROXIMAL_MUSCLE_WEAKNESS
HP_LIMB_MUSCLE_WEAKNESS

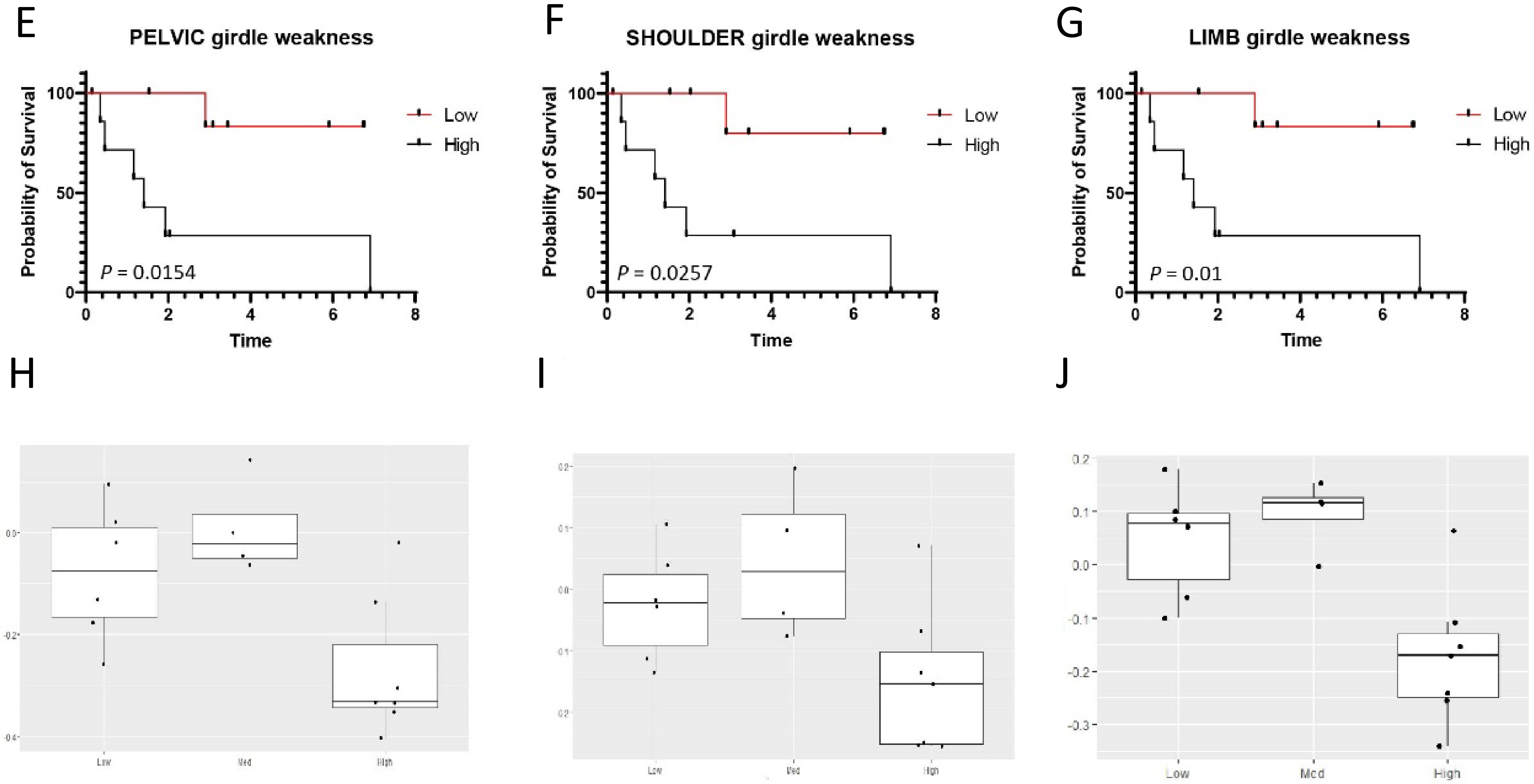

E
PELVIC girdle weakness
Probability of Survival
100
50
0
Time
0
2
4
6
8
Low
High
P = 0.0154
F
SHOULDER girdle weakness
Probability of Survival
Time
Low
High
P = 0.0257
G
LIMB girdle weakness
Probability of Survival
Time
Low
High
P = 0.01
H
I
J
0.2
0.1
0.0
-0.1
-0.2
-0.3
Low
Med
High

**Figure S8.** Muscle weakness in PRMS **A-D.** Unsupervised hierarchical clustering of gene sets related to skeletal muscle. **E.-G.** Pelvic, shoulder, and limb girdle muscle weakness scores were averaged for each patient and plotted for overall survival. Low: below the median average scores. High: above the median average scores. **H.-J**. Pelvic, shoulder, and limb girdle muscle weakness scores are significantly lower in PRMS samples with high immune infiltrate scores. Welch two-sample t-tests were used to determine significance.

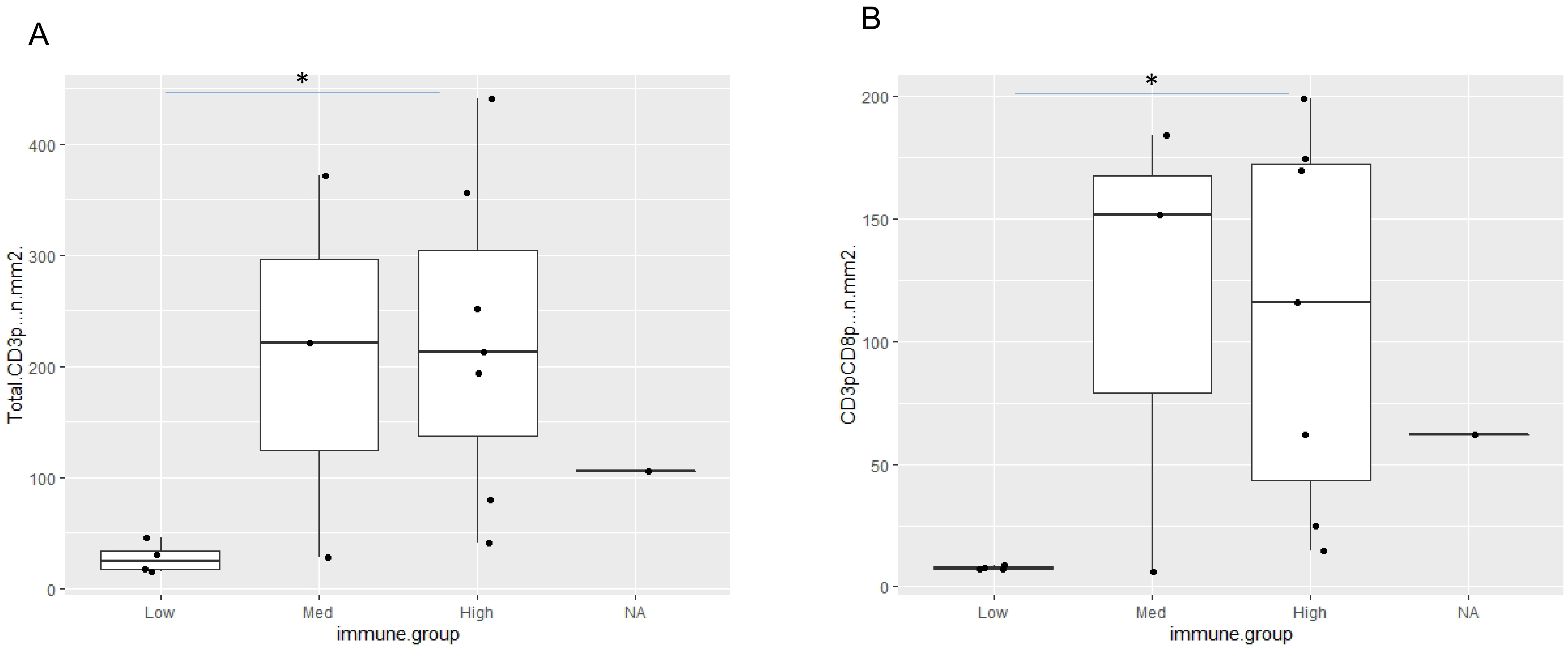
A
*
Total.CD3p...n.mm2.
0
100
200
300
400
Low
Med
High
NA
immune.group
B
*
CD3pCD8p...n.mm2.
0
50
100
150
200
Low
Med
High
NA
immune.group

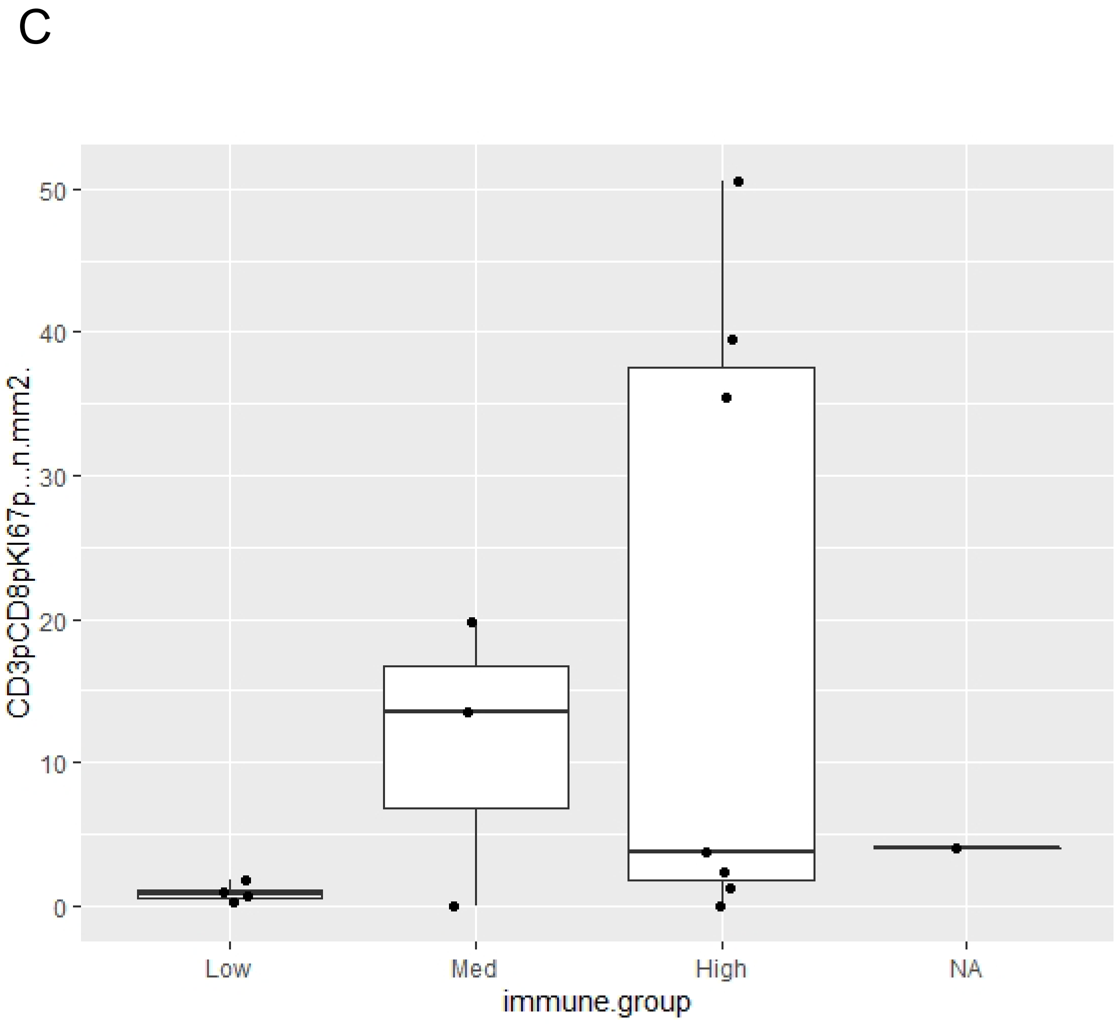
C
50
40
30
20
10
0
CD3pCD8pKI67p...n.mm2.
Low
Med
High
NA
immune.group

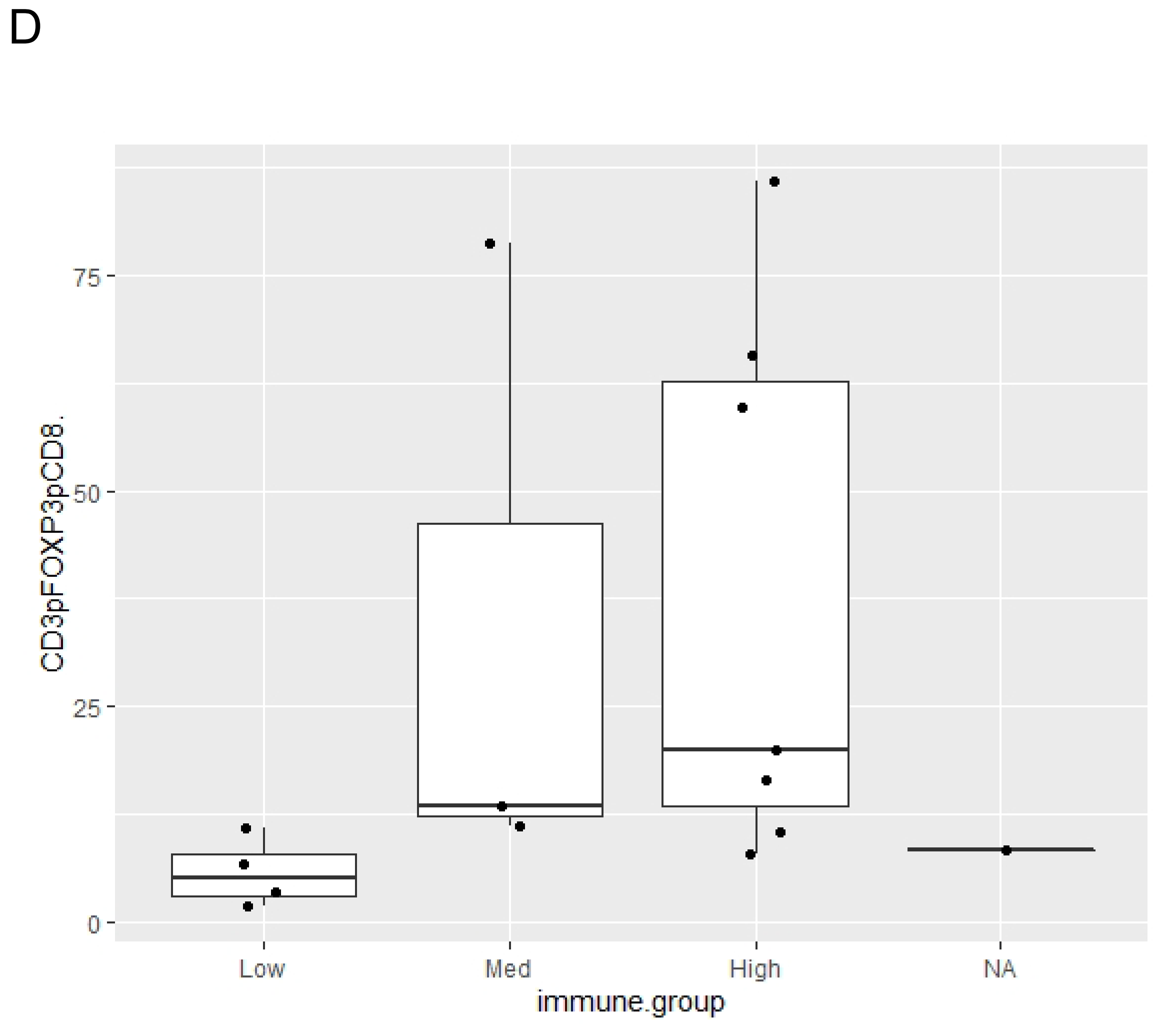
D
75
50
25
0
CD3pFOXP3pCD8.
Low
Med
High
NA
immune.group

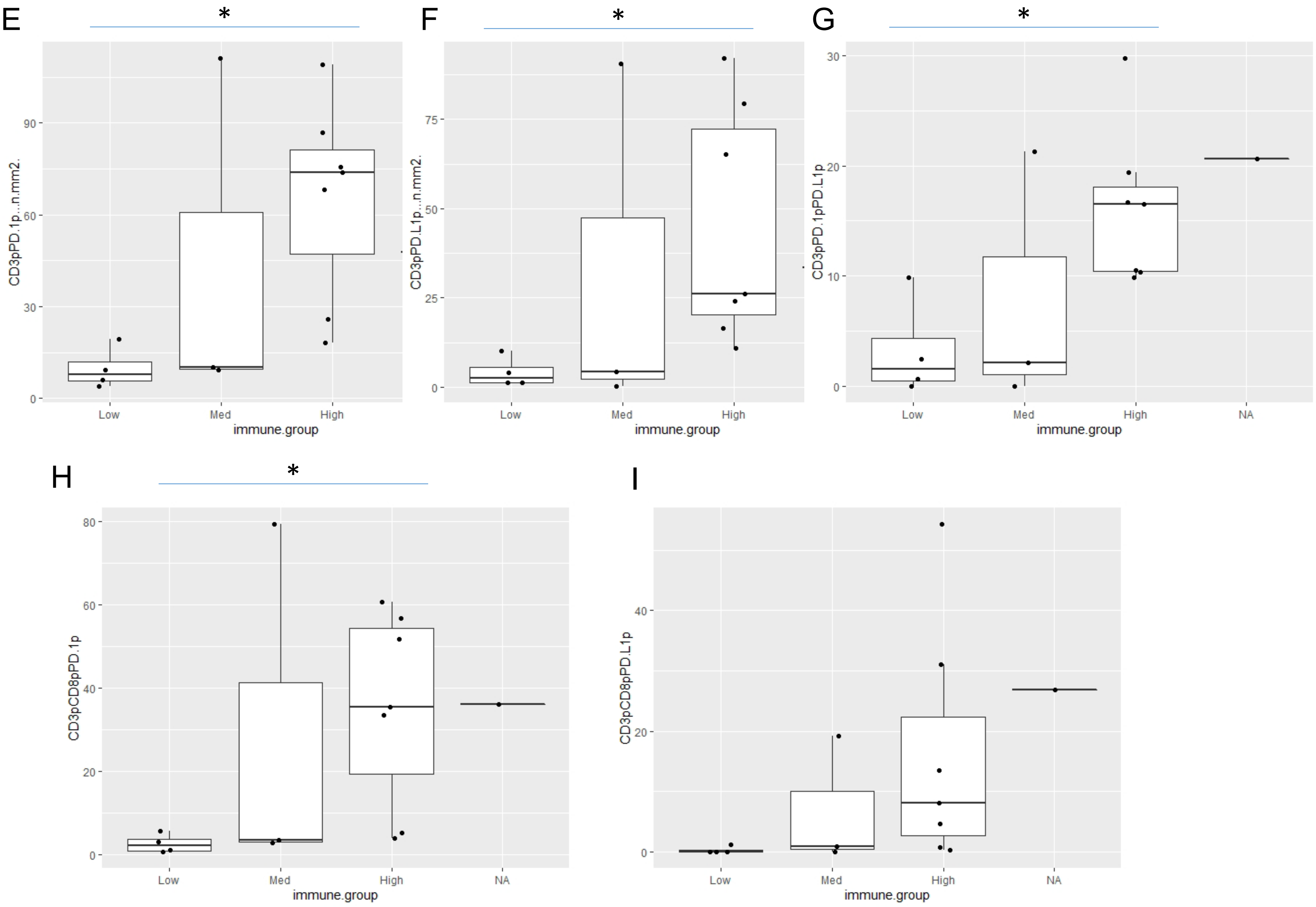
E
*
CD3pPD.1p...n.mm2.
0
30
60
90
Low
Med
High
immune.group
F
*
CD3pPD.L1p...n.mm2.
0
25
50
75
Low
Med
High
immune.group
G
*
CD3pPD.1pPD.L1p
0
10
20
30
Low
Med
High
NA
immune.group
H
*
CD3pCD8pPD.1p
0
20
40
60
80
Low
Med
High
NA
immune.group
I
CD3pCD8pPD.L1p
0
20
40
Low
Med
High
NA
immune.group

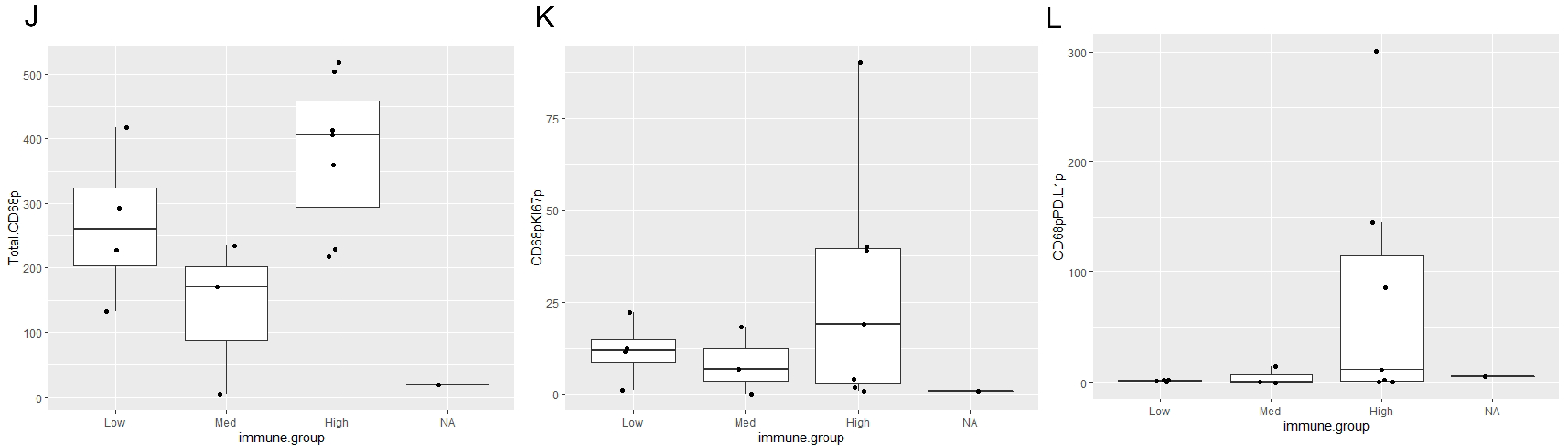


**Figure S9.** Validation of immune infiltrate by immunofluorescence. **A.** The total number of CD3+ staining cells (number per mm2, y-axis) according to the immune infiltrate group delineated by transcriptome data. **B.** Cytotoxic T-cells that are both CD3+ and CD8+ for each immune infiltrate group. **C.** Proliferating cytotoxic T-cells (CD3+/CD8+/Ki67+). **D.** Regulatory T-cells (CD3+/FOX3P+/CD8+. **E.-G.** T-cells with checkpoint expression (PD1+ and/or PDL1+). **H.-I.** CD3+CD8+ PD1+/PDL1+ cells. **J.-L.** CD68+cells. Immune groups based on Fig. 7A: Low = low; Med = medium; High = high; NA = RNA data not available. Welch Two Sample t-tests.

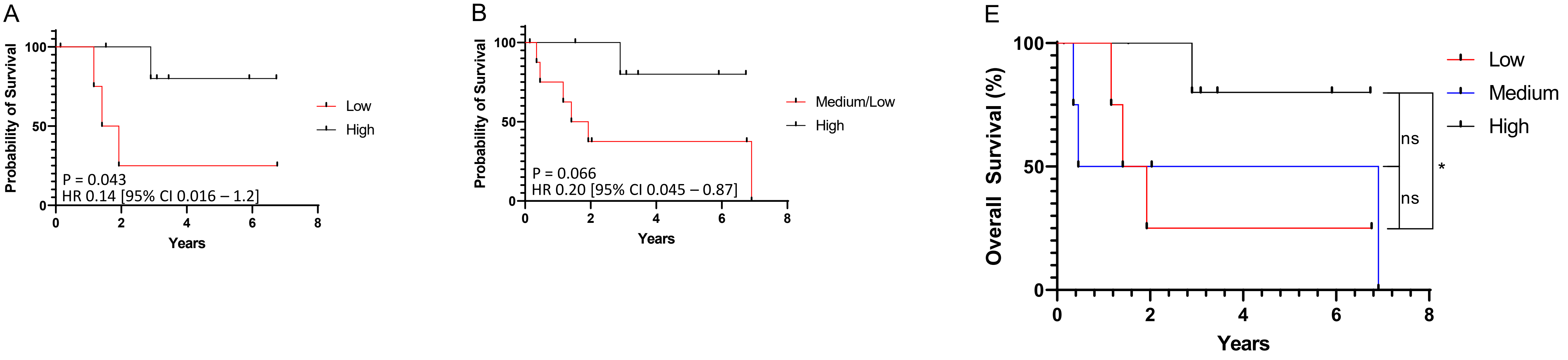


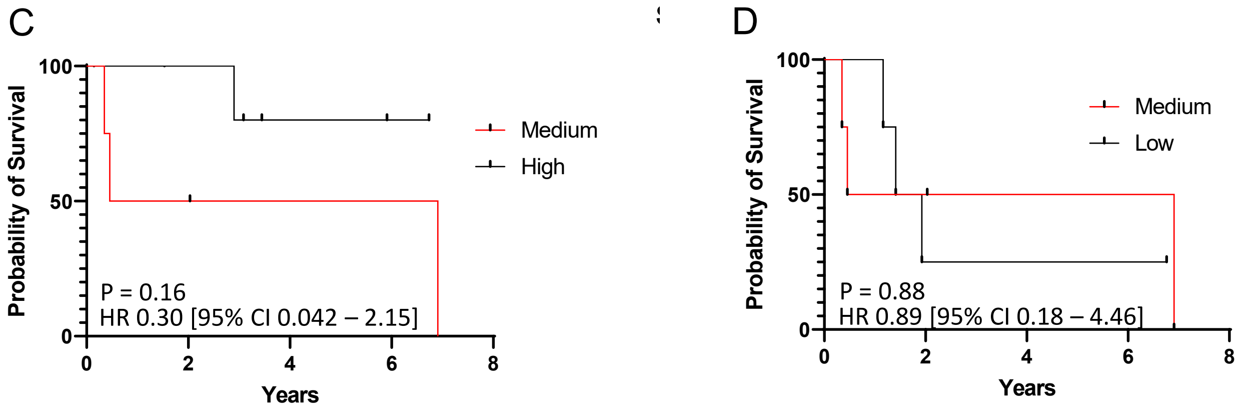


**Figure S10**. Kaplan-Meier curves for 15 PRMS patients separated according to their immune infiltrate classification: Low (L), Medium (M), and High (H). HR = hazard ratio; ns = not significant; * = p-value < 0.05 calculated by log rank tests.

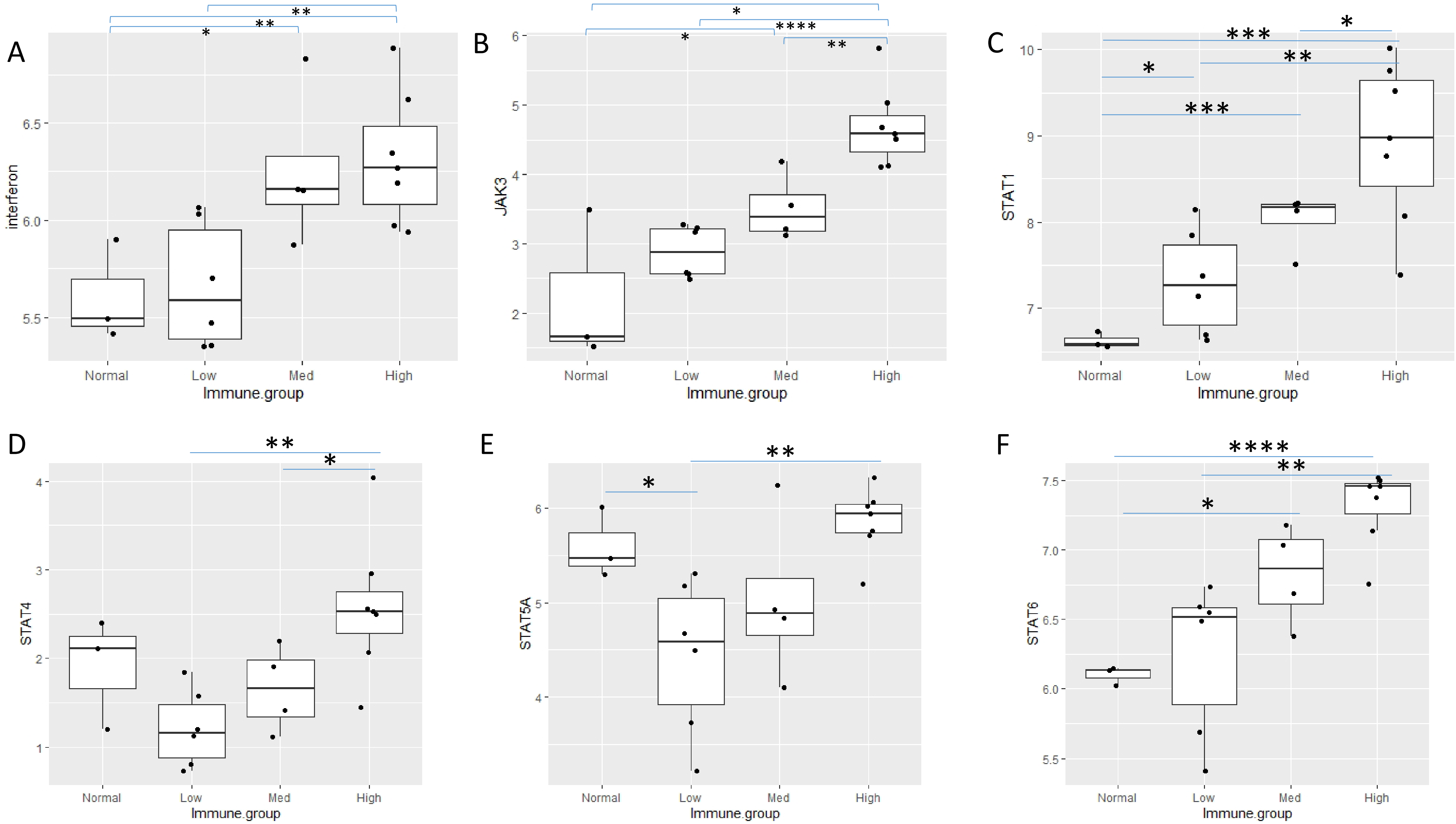
A
interferon
Immune.group
B
JAK3
Immune.group
C
STAT1
Immune.group
D
STAT4
Immune.group
E
STAT5A
Immune.group
F
STAT6
Immune.group
Normal
Low
Med
High

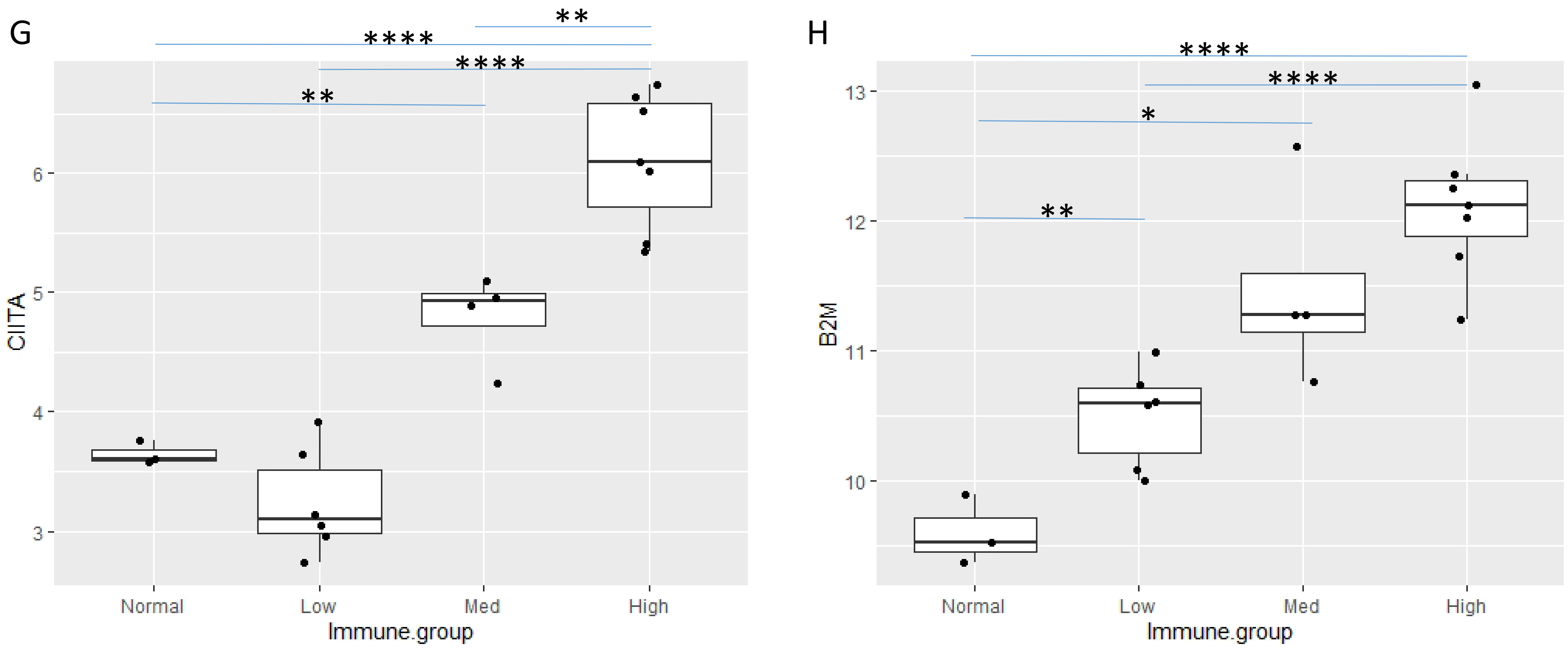


**Figure S11.** Differentially expressed genes among immune infiltrate groups that contribute to immunosuppression. **A.** All interferon genes combined into a gene set (interferon) **B.-F.** JAK-STAT signaling. **G.-H.** Antigen-presentation controllers *CIITA* and *B2M*. Welch two sample t-tests were used to determine significance.

**Figure S12.** Immunosuppressive molecules **A.-C.** Immune evasion **D.** Inhibitor of T-cell activation. Welch two sample t-tests were used to determine significance.

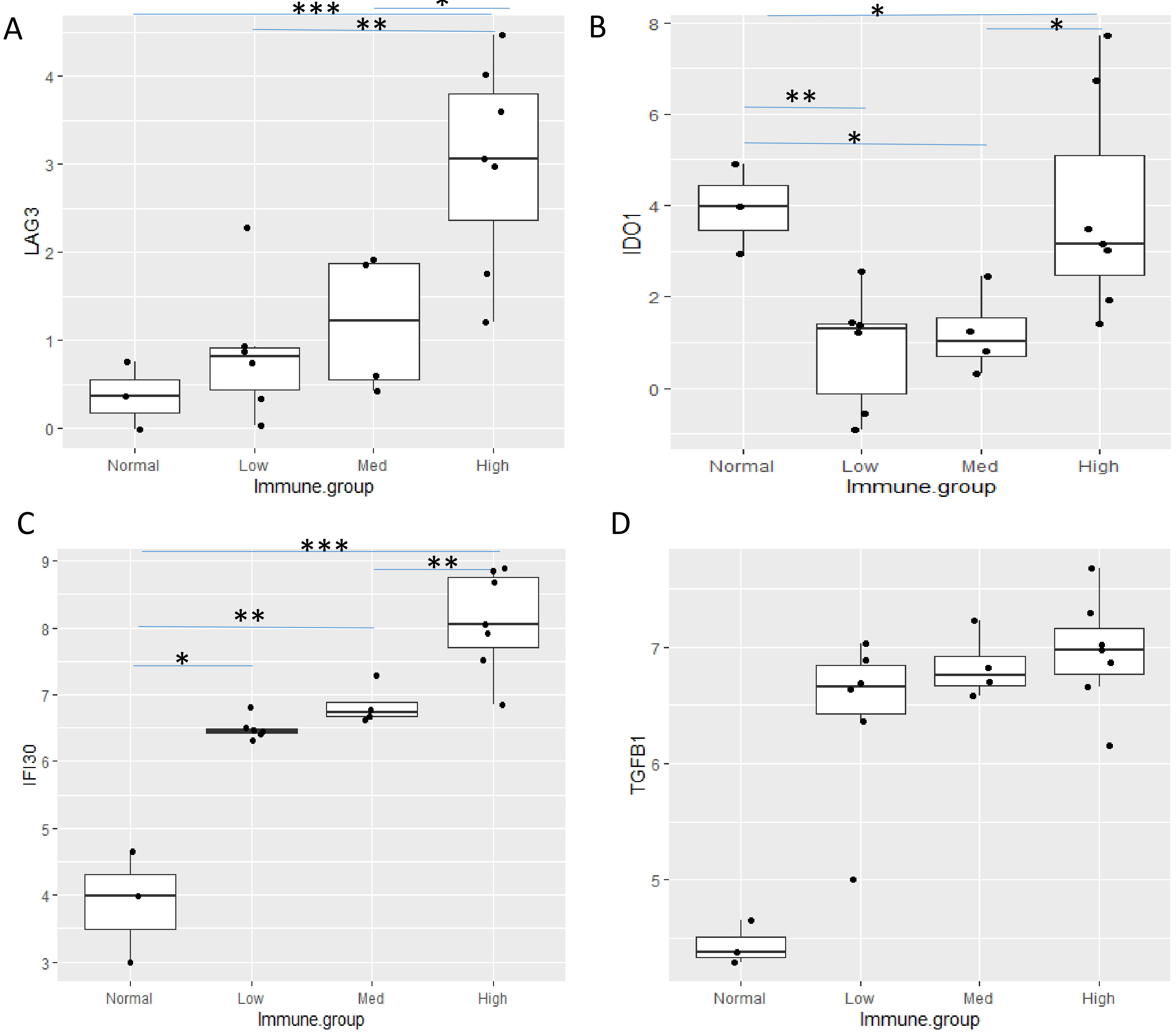

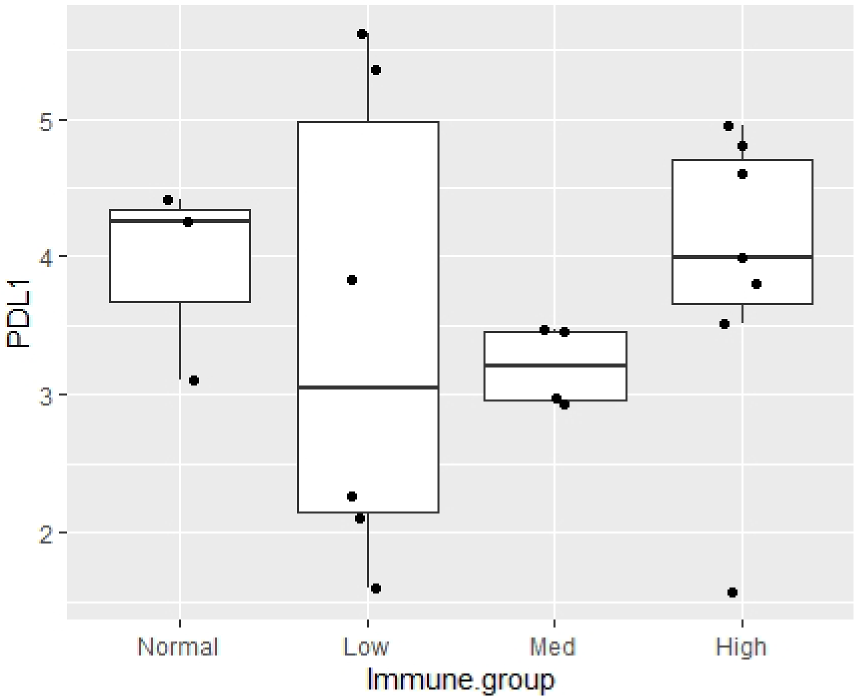


**Figure S13.** PDL1 is not differentially expressed according to immune group.

**Supplemental Methods**

**Single Sample Gene Set Enrichment Analysis:** We first calculated enrichment scores for each pairing of a sample and a pathway gene set in the MSigDB database. Each ssGSEA enrichment score can evaluate the genes in a pathway for their distribution in an ordered gene list ranked by expression of all the genes in a sample, which represents the degree to which the genes in the gene set are coordinately up- or down-regulated within a sample. We then compared the ssGSEA enrichment scores of pathway gene sets across various different types of sarcoma samples using principal component analysis, hierarchical clustering of samples, and differential analysis. The normalized ssGSEA pathway scores of the patient samples were submitted for unsupervised clustering.

**Methylation array analyses:** Probes were removed based on these criteria (*53*): 1) poor performing probes with detection $p \geq 0.01$; 2) the probes on the sex chromosomes (X and Y); 3) the probes that are known to have common SNPs at the CpG site; and 4) the probes that have shown to be cross-reactive and mapping to multiple places in the genome. Between-sample normalization was then performed using the preprocessQuantile function in the minfi R package, and normalized intensity values were then converted into M values, which were used in the downstream analyses: principal component analysis, hierarchical clustering of samples, and differential methylation analysis.

**Comparison of PRMS profiles with profiles of other tumor types**

We compared the genetic, epigenetic, and immune profiles of our PRMS samples to those of ARMS, ERMS, and other complex-karyotype (complex) sarcomas, including osteosarcoma, undifferentiated pleomorphic sarcoma (UPS), leiomyosarcoma, myxofibrosarcoma, and pleomorphic liposarcoma, as well as an independent PRMS cohort from Delespaul et al. (GSE75885) (PMID: 27528700). We also determined the degrees to which the immune profiles of PRMS were similar to those of other solid tumors from The Cancer Genome Atlas (TCGA).
